# Host–pathogen associations revealed by genotyping of European strains of *Anaplasma phagocytophilum* to describe natural endemic cycles

**DOI:** 10.1186/s13071-023-05900-3

**Published:** 2023-08-16

**Authors:** Julia Fröhlich, Susanne Fischer, Benjamin Bauer, Dietmar Hamel, Barbara Kohn, Marion Ahlers, Anna Obiegala, Evelyn Overzier, Martin Pfeffer, Kurt Pfister, Cristian Răileanu, Steffen Rehbein, Jasmin Skuballa, Cornelia Silaghi

**Affiliations:** 1 Comparative Tropical Medicine and Parasitology, Ludwig-Maximilians-Universität München, Leopoldstrasse 5, 80802 Munich, Germany; 2 Institute of Infectology, Friedrich-Loeffler-Institut, Federal Research Institute for Animal Health, Südufer 10, 17943 Greifswald-Insel Riems, Germany; 3 Clinic for Swine and Small Ruminants, Forensic Medicine and Ambulatory Service, University of Veterinary Medicine Hannover, Foundation, Bischofsholer Damm 15, 30173 Hannover, Germany; 4 Boehringer Ingelheim Vetmedica GmbH, Kathrinenhof Research Center, Walchenseestr. 8-12, 83101 Rohrdorf, Germany; 5 Clinic for Small Animals, Department of Veterinary Medicine, Freie Universität Berlin, Oertzenweg 19B, 14163 Berlin, Germany; 6 agro prax GmbH, Werner-von-Siemens-Str. 2, 49577 Ankum, Germany; 7 Institute of Animal Hygiene and Veterinary Public Health, University of Leipzig, An den Tierkliniken 1, 04103 Leipzig, Germany; 8 Chemical and Veterinary Investigations Office Karlsruhe (CVUA Karlsruhe), Weissenburger Str. 3, 76187 Karlsruhe, Germany

**Keywords:** *Anaplasma phagocytophilum*, Epidemiological cycles, Host-variant combinations, Sequence networks

## Abstract

**Background:**

The zoonotic intracellular alpha-proteobacterium *Anaplasma phagocytophilum* is a tick-transmitted pathogen. The associations between vertebrate reservoirs and vectors are described as wide-ranging, and it was previously shown that the pathogenicity of *A. phagocytophilum* differs depending on the combination of pathogen variant and infected host species. This leads to the question of whether there are variations in particular gene loci associated with different virulence. Therefore, this study aims at clarifying existing host-variant combinations and detecting possible reservoir hosts. To understand these interactions, a complex toolset for molecular epidemiology, phylogeny and network theory was applied.

**Methods:**

Sequences of up to four gene loci (*msp4*, *msp2*, *groEL* and *16S* rRNA) were evaluated for different isolates from variable host species, including, for example, dogs, cattle and deer. Variant typing was conducted for each gene locus individually, and combinations of different gene loci were analysed to gain more detailed information about the genetic plasticity of *A. phagocytophilum*. Results were displayed as minimum spanning nets and correlation nets.

**Results:**

The highest diversity of variants for all gene loci was observed in roe deer. In cattle, a reduced number of variants for *16S* rRNA [only 16S-20(W) and 16S-22(Y)] but multiple variants of *msp4* and *groEL* were found. For dogs, two *msp4* variants [m4-20 and m4-2(B/C)] were found to be linked to different variants of the other three gene loci, creating two main combinations of gene loci variants. Cattle are placed centrally in the minimum spanning net analyses, indicating a crucial role in the transmission cycles by possibly bridging the vector-wildlife cycle to infections of humans and domestic animals. The minimum spanning nets confirmed previously described epidemiological cycles of the bacterium in Europe, showing separation of variants originating from wildlife animals only and a set of variants shared by wild and domestic animals.

**Conclusions:**

In this comprehensive study of 1280 sequences, we found a high number of gene variants only occurring in specific hosts. Additionally, different hosts show unique but also shared variant combinations. The use of our four gene loci expand the knowledge of host–pathogen interactions and may be a starting point to predict future spread and infection risks of *A. phagocytophilum* in Europe.

**Graphical abstract:**

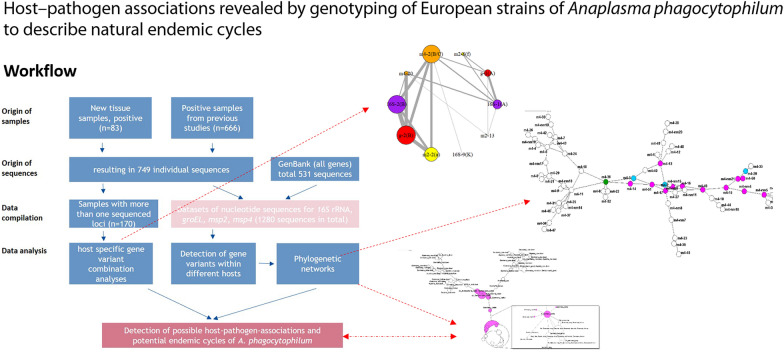

**Supplementary Information:**

The online version contains supplementary material available at 10.1186/s13071-023-05900-3.

## Background

*Anaplasma phagocytophilum* is a zoonotic intracellular alpha-proteobacterium transmitted by ixodid ticks. The main vector in Europe is the exophilic hard tick *Ixodes ricinus* [1]. *Anaplasma phagocytophilum* causes granulocytic anaplasmosis in humans, horses, dogs and cats and tick-borne fever (TBF) in ruminants [2, 3]. Within *I. ricinus*, transstadial but not transovarial transmission has been described [4]. Thus, the transmission dynamics of *A. phagocytophilum* predominantly rely on horizontal transmission between ticks and vertebrate hosts [5]. This also indicates that there are complex evolutionary pressures on *A. phagocytophilum*, as the interactions between vertebrate reservoirs and vectors are numerous, and also has a impact on the number of genetic variants found in *A. phagocytophilum* [6]. To date, it remains a challenge to unravel the complex epidemiological cycles of *A. phagocytophilum* and to define reservoir hosts [5]. In general, the distinction between *A. phagocytophilum* strains has been focused on host level and specific genetic loci, such as the *16S* ribosomal RNA gene (*16S* rRNA). A division in genetic variants derived from ruminant and non-ruminant hosts was proposed previously and confirmed by several studies [7–10]. Studies on the spatial distribution of *A. phagocytophilum* genotypes have also been conducted; for example, in Asia a common cycle for ruminant livestock and small rodents based on the *16S* rRNA and the partial *p44ESup1* genes was proposed [11]. Spatial studies carried out in North America detected two main variants, also based on the *16S* rRNA of *A. phagocytophilum* [12, 13], with both strains differing in their pathogenicity for humans. While white-footed mice *(Peromyscus leucopus)* are suspected to carry the human pathogenic strain AP-ha, white-tailed deer *(Odocoileus virginianus)* are suspected to carry variant AP-Variant-1 which is less pathogenic to humans [7, 14]. In Europe, the epidemiological cycles are not completely understood. Hosts such as red deer (*Cervus elaphus*) and roe deer (*Capreolus capreolus*) have been suspected to be reservoir hosts of *A. phagocytophilum*, but are not yet clearly identified [5, 15]. A European study suggested two separate cycles for *A. phagocytophilum* in Europe, namely driven by ticks and either rodents or ruminants, on the basis of analysing the *16S* rRNA, major surface protein 4 (*msp4*) and the *DOV1* (a noncoding region) gene [16]. The results of a study from Jahfari et al. [17] suggested four geographically distinct ecotypes of *A. phagocytophilum* in Europe based on comparison of *groEL* (heat shock protein) sequences. This finding was further developed in a global approach by Jaarsma et al. [6], confirming the four previously distinguished ecotypes from wild and domestic animals, among others from European hedgehogs, wood mice, mouflons, sheep, roe deer, badgers and foxes [6, 17]. The authors of former studies concluded that the sequence variability at one locus might not be sufficient to determine the genetic diversity of a certain *A. phagocytophilum* strain in total [18, 19]. Multilocus sequence typing with a different number of loci was previously conducted to gain more detailed information on those pathogens [10, 15, 20, 21]. By analysing partial *16S* rRNA, *groEL*, *msp4* and/or *msp2* (major surface protein 2) genes of *A. phagocytophilum*, our group previously provided information on diverse variants of these genes occurring in multiple animal species in Europe, such as goats, cattle, horses, dogs, wild ungulates and hedgehogs [22–26].

This study aims to summarize all of these previous reports and to present new approaches for analysing samples of *A. phagocytophilum* in Europe. Therefore, we (i) genotyped and characterized samples of the four partial genes (*16S* rRNA, *groEL*, *msp4*, *msp2*) of *A. phagocytophilum* from different mammalian host animals and (ii) conducted network analyses on the relationships of *A. phagocytophilum* variants derived from different host species.

## Methods

### Dataset composition

**Fig. 1:**
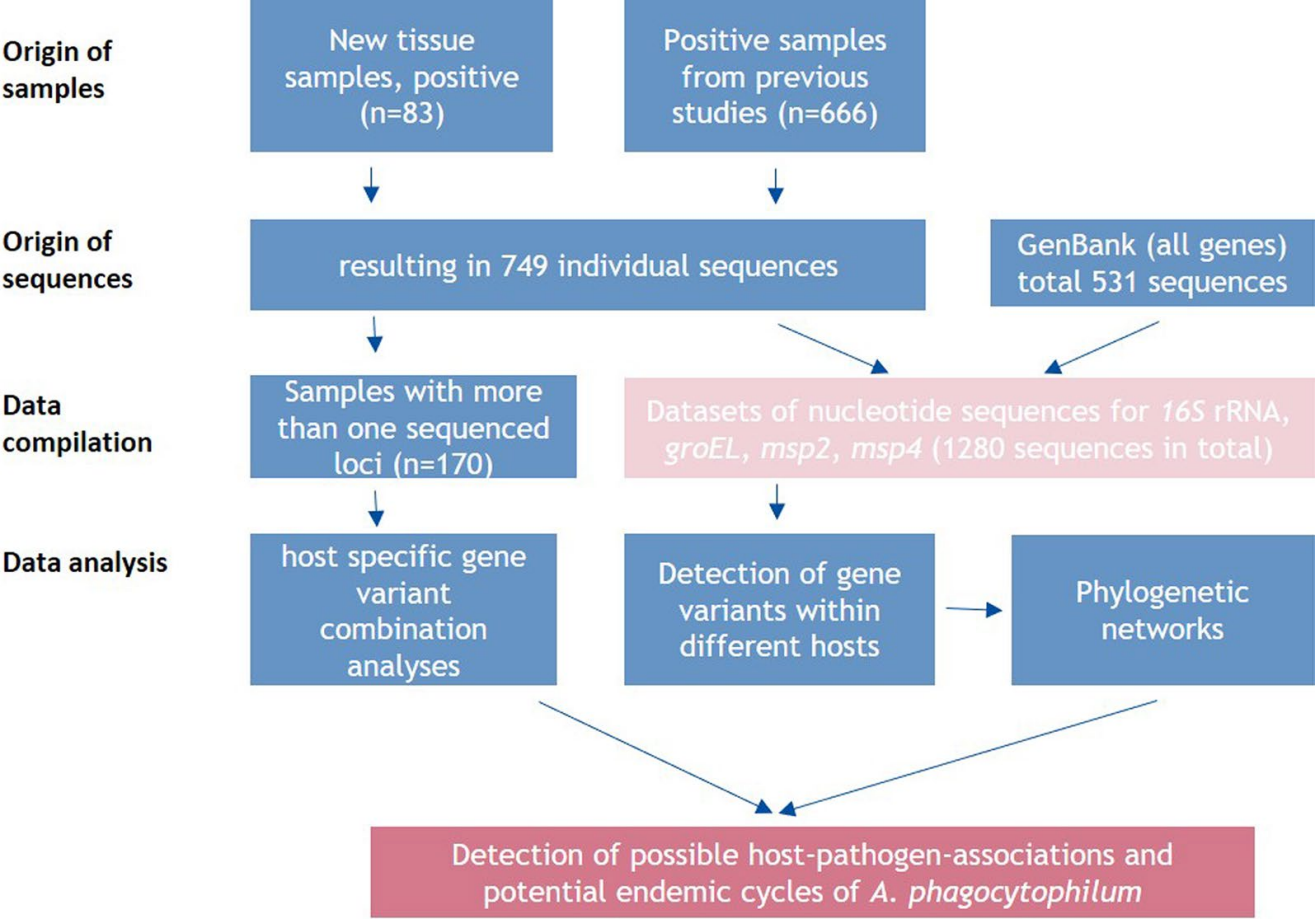
Workflow for the data sampling, preparation, sequencing, compilation and analysis steps performed in the present study. *groEL*, Heat shock protein operon; *16S* rRNA, *16S* ribosomal RNA gene, *msp4*/*msp2*, major surface protein 2/major surface protein 4 genes

Over a period of more than 13 years, a large dataset of sequences of the four partial genes (*16S* rRNA, *msp2*, *msp4* and *groEL*) of *A. phagocytophilum* has been obtained, which includes a wide range of host species. This dataset was compiled applying three different strategies, with samples originating from (i) positive samples detected within the present or (ii) previous studies from our group and (iii) from additional sequences downloaded from GenBank. In general, all wildlife samples were screened for *A. phagocytophilum*, and all livestock and companion animals were investigated based on a clinical suspicion or epidemiological link to confirmed cases of anaplasmosis. We obtained a total of 1280 sequences (Fig. 1). All accession numbers of the included sequences can be found in Additional file 1: Table S1.

### DNA extraction, PCR and sequencing

A total of 356 tissue samples from wild animals were available for screening in this study. From each sample from wild ruminants, we extracted 10–15 g of spleen (in 1 case, the liver; see Table 1) using the High Pure PCR Template Preparation Kit® (Roche Diagnostics GmbH, Mannheim, Germany), and from each sample from red foxes, we extracted DNA from spleen samples using the QIAmp DNA Mini Kit (Qiagen, Hilden, Germany), following the respective manufacturer’s protocol in both cases. Quality and quantity of the extracted DNA were measured on a full-spectrum (220–750 nm) spectrophotometer (NanoDrop® ND-1000; PeqLab Biotechnologie GmbH, Erlangen, Germany). For further use in PCR assays, the extracted DNA was diluted to a concentration of < 130 ng/μl to prevent false negatives. All PCR assays were performed as described in this section. Primers and probes are shown in Additional file 2: Table S2.

**Table 1:** *Anaplasma phagocytophilum*-positive spleen samples detected by real-time PCR in the present study

Animal species	Number of samples	Number of positive samples (%)
Roe deer	16	13 (81.3)
Red deer^a^	37	13 (35.1)
Sika deer	17	13 (76.5)
Fallow deer	7	5 (71.4)
Chamois	7	4 (57.1)
Red fox	260	34 (13.1)
Wild boar	12	1 (8.3)
Total	356	83 (23.3)

^a^One of these samples was from the liver (tested positive), not the spleen

All 356 DNA extracts from this study were screened for a fragment of the *msp2* gene by real-time PCR [27].

For all positive samples, Sanger sequencing was performed following the different PCR assays. For partial *16S* rRNA gene sequences, a nested PCR with a final amplicon size of 546 bp [28] was used, of which 497 bp was used for variant definition. For the *groEL* gene sequences, a hemi-nested PCR was performed according to Alberti et al. [29], with a resulting amplicon of 573 bp in length, of which 530 bp were used for variant comparison analyses. A nested PCR was also used for targeting the partial *msp4* gene according to Bown et al. [18], with a 340-bp amplicon used for analyses in the present study. To target the partial *msp2* gene (893 bp), we used a conventional PCR assay [30]. All PCR products were purified with the QIAquick PCR Purification Kit® (Qiagen) according to the manufacturer’s instructions. Quality and quantity of the purified PCR products were determined with a spectrophotometer (NanoDrop® ND-1000; PeqLab Biotechnologie GmbH). After purification, sequencing of the PCR amplicons was performed by Eurofins Genomics (Ebersberg, Germany). In the case of nested PCR protocols, the product of the second PCR and the inner primers were chosen for sequencing. The chromatograms of the sequences were analysed and evaluated with Chromas Lite® (Technelysium Pty. Ltd, South Brisbane, Australia; http://www.technelysium.com.au). The forward and reverse sequences of the samples were assembled and a contiguous sequence was generated.

### GenBank data

For spanning net analyses, genetically comparable sequences from the NCBI database GenBank (http://www.ncbi.nlm.nih.gov/) were chosen and downloaded by screening the database for “*Anaplasma phagocytophilum*” and the four genes examined in this study. The selection included sequences from the same gene region and of the same length as the newly sequenced samples. Altogether, 531 sequences with known animal species origin were downloaded from GenBank and used for comparison. The sequences originated from Europe, North America and Asia.

### Data compilation and naming of sequences

For every partial gene, the sequences determined in this study were named according to the abbreviation of the respective partial genes (*16S* rRNA: “16S-”, *groEL*: “g-”, *msp2*: “m2-”, *msp4*: “m4-”) and a numerical sequence. The letter in brackets refers to a nomenclature given in previous studies by our group [22–24, 31–33]. The pre-processed alignment was conducted with ClustalW [34] in MegaX [35]. For ease of analysis, nucleotide sequences from GenBank that did not match with a sequenced variant from the above-mentioned previous studies, were considered as “additional” variants and newly named (“16S-nm”, “g-nm”, “m4-nm”, “m2-nm”), where the letters “nm” stand for “no match”; in addition, they were also enumerated (e.g. “16S-nm1”).

### Data analyses

Net graphics were conducted with R [36] and were created under the R packages igraph [37] and ggplot2 [38]. Minimum spanning nets for distance were constructed in SplitsTree [39, 40] under a Kimura-2-parameter model [41]. This evolutionary analysis produces connections of the input sequences without introducing additional ancestral nodes.

## Results

### Detection of host-gene variant combinations

In total we analysed 749 sequences extracted from our tissue samples and 531 sequences from additional samples in GenBank (Fig. 1). Of these 1280 sequences, 89 *msp2*, 692 *16S* rRNA, 234 *msp4* and 265 *groEL* were analysed. We found 40 different *msp2* variants in 14 host species, 51 different *16S* rRNA variants in 20 host species, 72 different *msp4* variants in 18 host species and 67 different *groEL* variants in 20 host species.

For the 749 sequences originating from our own tissue samples group, we obtained a total of 16 *msp2,* 23 *16S* rRNA, 50 *msp4* and 33 *groEL* variants; for 52 samples we were able to sequence all four genes (Additional file 2: Table S3). At 98.4%, the variants from the *16S* rRNA locus have the highest similarity in this dataset, followed by 95.9% for *groEL*, 87.9% for *msp4* and only 67.1% for *msp2*. Most samples from the previous studies performed by our group (*n* = 428) originated from Germany. From the newly generated sequences, we obtained sequences for all four gene loci of 52 *A. phagocytophilum*-positive samples. For an additional 48 and 70 samples, three and two different gene loci, respectively, were sequenced. Details can be found in Additional file 2: Table S3. For the phylogenetic network analyses, the complete dataset of different variants per loci were used. For the 531 GenBank sequences, the origin was also known, with *16S* rRNA and *msp2* sequences originating predominately from the USA, while most *groEL* and *msp4* sequences originated from Europe.

### Occurrence of gene variant combinations

**Fig. 2:**
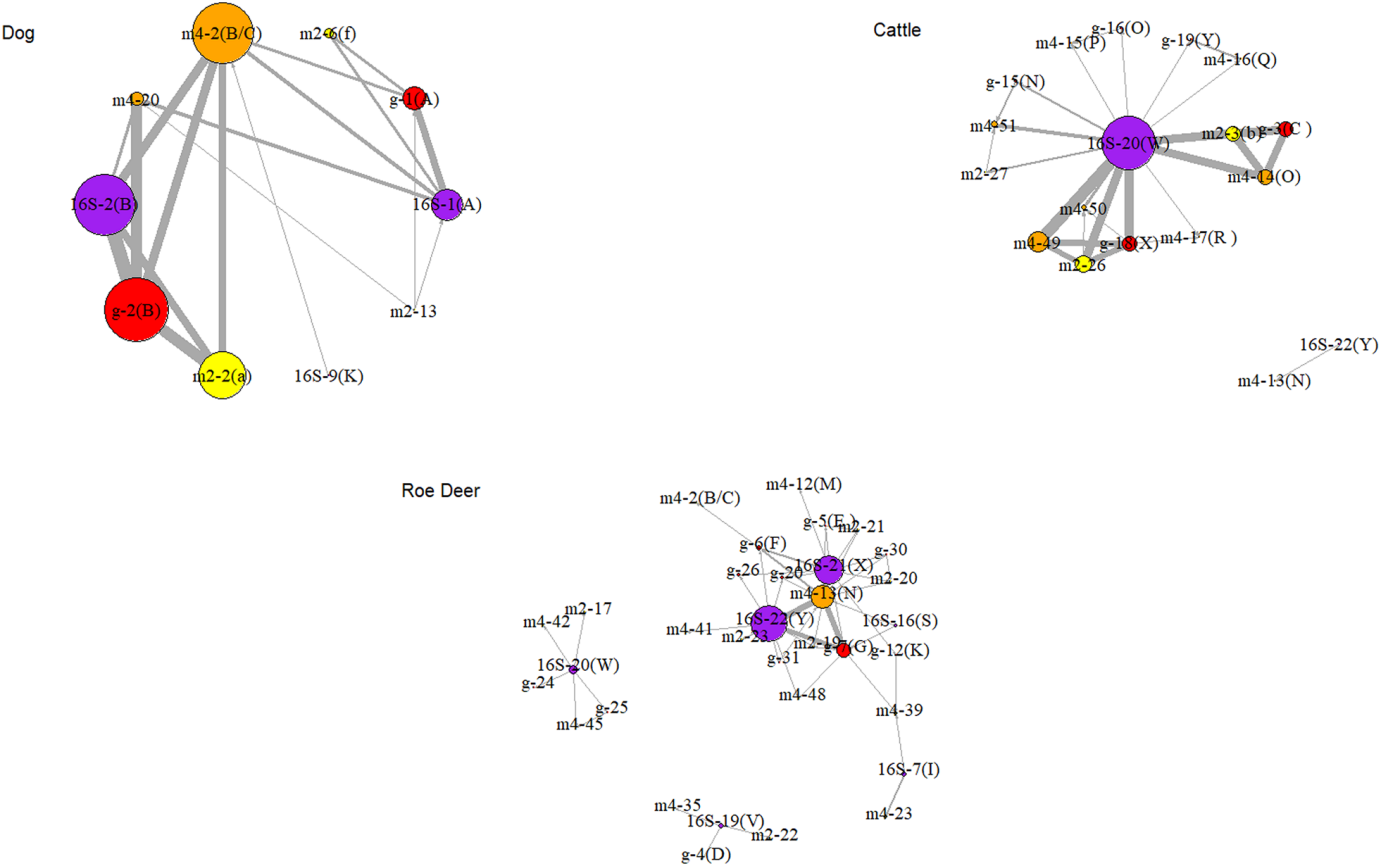
The net for all connections of all variants of the four sequenced genes. Node’ diameters correlate with the number of sequences of the indicated variant in the samples. Colours indicate each of the different genes analysed, with purple indicating the *16S* rRNA variants; yellow, *msp2* variants; orange, *msp4* variants; and red, *groEL* variants. The width of the connecting lines represents the total number of specific connections for dog, cattle and roe deer, respectively. Labelling is explained in “Data compilation and naming of sequences” section

To show all known connections of variants of all four sequenced genes within individual hosts the data subset from Additional file 2: Table S3 was used. Analyses were conducted for dog, cattle and roe deer (Fig. 2).

For dogs, two different *msp4* variants (m4-20 and m4-2(B/C)) occurred (Fig. 2). 16S-2(B), g-2(B) and m2-2(a) were directly connected to each other and to no other variants of these genes, but they were connected to both *msp4* variants. 16S-1(A) and g-1(A) were also specifically combined. We observed that the 16S-1(A) variant also occurred in combination with the two different major surface proteins [*msp4*: m4-2(B/C), m4-20; *msp2*: m2-6(f)] in six dogs, but also in one horse, 26 hedgehogs and two foxes (Additional file 2: Table S3). Samples from cattle were similar, with two distinct sequence variant combinations in 15 examined animals, but other combinations also occurred. All available variant combinations from cattle are shown in Fig. 2. Variant 16S-20(W) can be seen as the central connecting point; the only other *16S* rRNA variant (16S-22(Y)) was located outside the main net and connected only with a *msp4* variant (m4-13(N)) which connected to neither 16–20(W) nor the main net. Two different combinations of the other three genes can be detected [16S-20(W)/m2-3(b)/m4-14(O)/g-3(C) and 16S-20(W)/m2-26/m4-49/g-18(X)]. Wild animals, such as, for example, roe deer (Fig. 2), show a more diverse pattern of connections than the data presented for cattle and dogs. The most common variants of *16S* rRNA, 16S-22(Y) and 16S-21(X), were both connected to the most frequent *msp4* variant [m4-13(N)]. The combination of 16S-22(Y), m4-13(N) and g-7(G) is the only one occurring more than once, but multiple single other variants and combinations can be detected.

### Phylogenetic networks

Phylogenetic networks were calculated to reveal the relationship between the individual variants of each gene. To this end, exemplary sequences for each variant were chosen (Additional file 2: Tables S4, S5, S6, S7) and correlated with the related hosts. A list of all available *msp2* variants and their host allocation can be found in Additional file 2: Table S4. Evaluation of *msp2* sequences showed that variants from European ruminants do not match with GenBank sequences from the USA, with the exception of three sequences (Accession numbers: AY706393, AY706392, AY706393) [44–46].

**Fig. 3:**
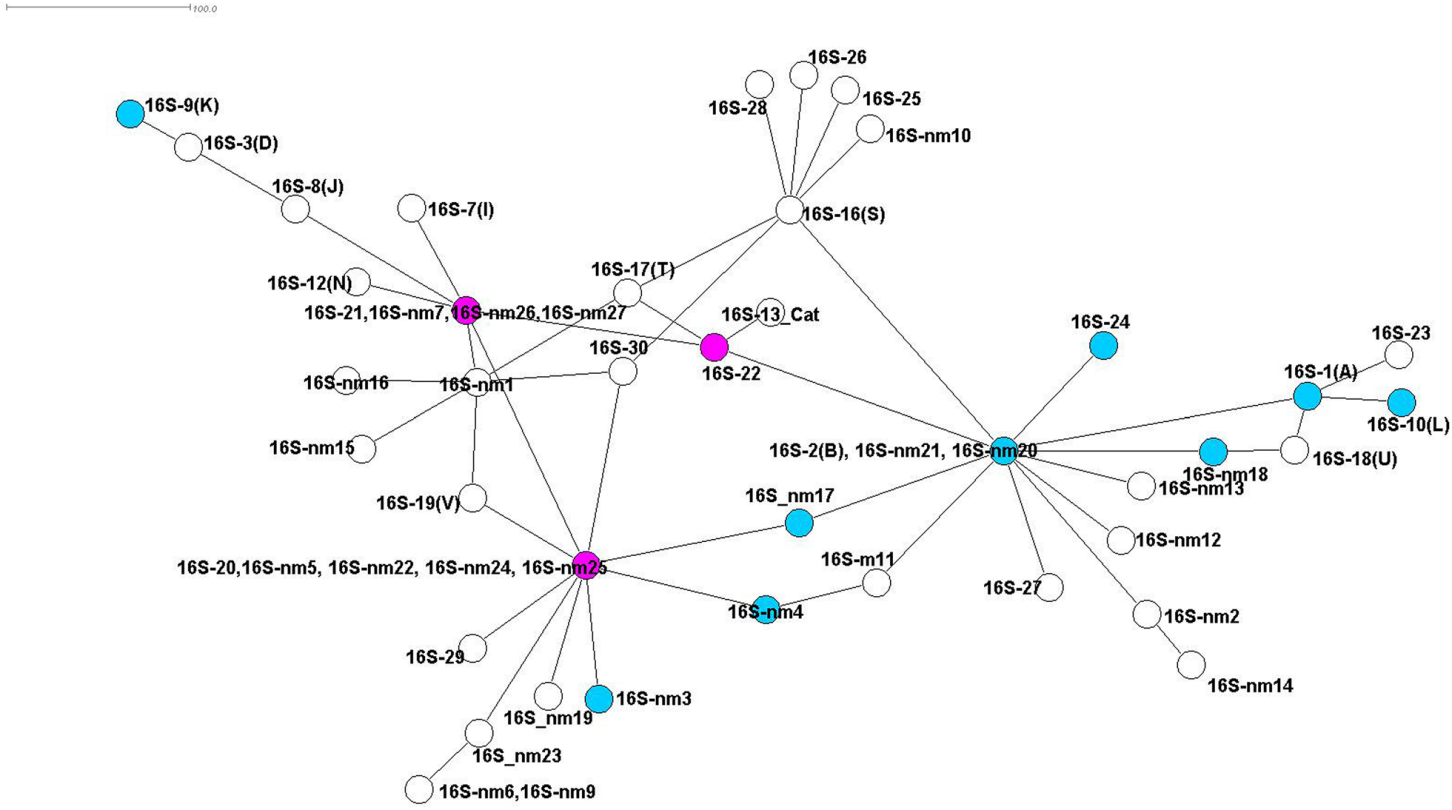
Minimum spanning net for distances on basis of a Kimura two-parameter (K2P) model corrected distance matrix from sequence alignment, showing the relations between the different *A. phagocytophilum 16S* rRNA variants determined from this study. The corresponding hosts and an exemplary sequence for each variant are given in Additional file 2: Table S5. Variants highlighted in pink include cattle as host, and those highlighted in blue include dogs as host. Circles with multiple names represent variants separated only by 1 ambiguous nucleotide. Labelling is explained in “Data compilation and naming of sequences” section

For *16S* rRNA, 23 different variants were detected and combined with variants known from previous data (16S-nm), resulting in a total of 51 variants (Fig. 3; Additional file 2: Table S5). Each node represents one or multiple of these 51 variants, and the lines show its relative distance to the genetically nearest relative. The net is connected by inner nodes directly or indirectly connected to one another. All cattle *16S* rRNA variants (marked in pink in Fig. 3), i.e. 16S-20(W), 16S-22(Y) and 16S-21(X), are part of the inner net. In dogs, the *16S* rRNA variants (marked in blue) show a high variability. Roe deer variant sequences are distributed throughout the complete net, mostly directly or indirectly connected to one of the cattle variants.

**Fig. 4:**
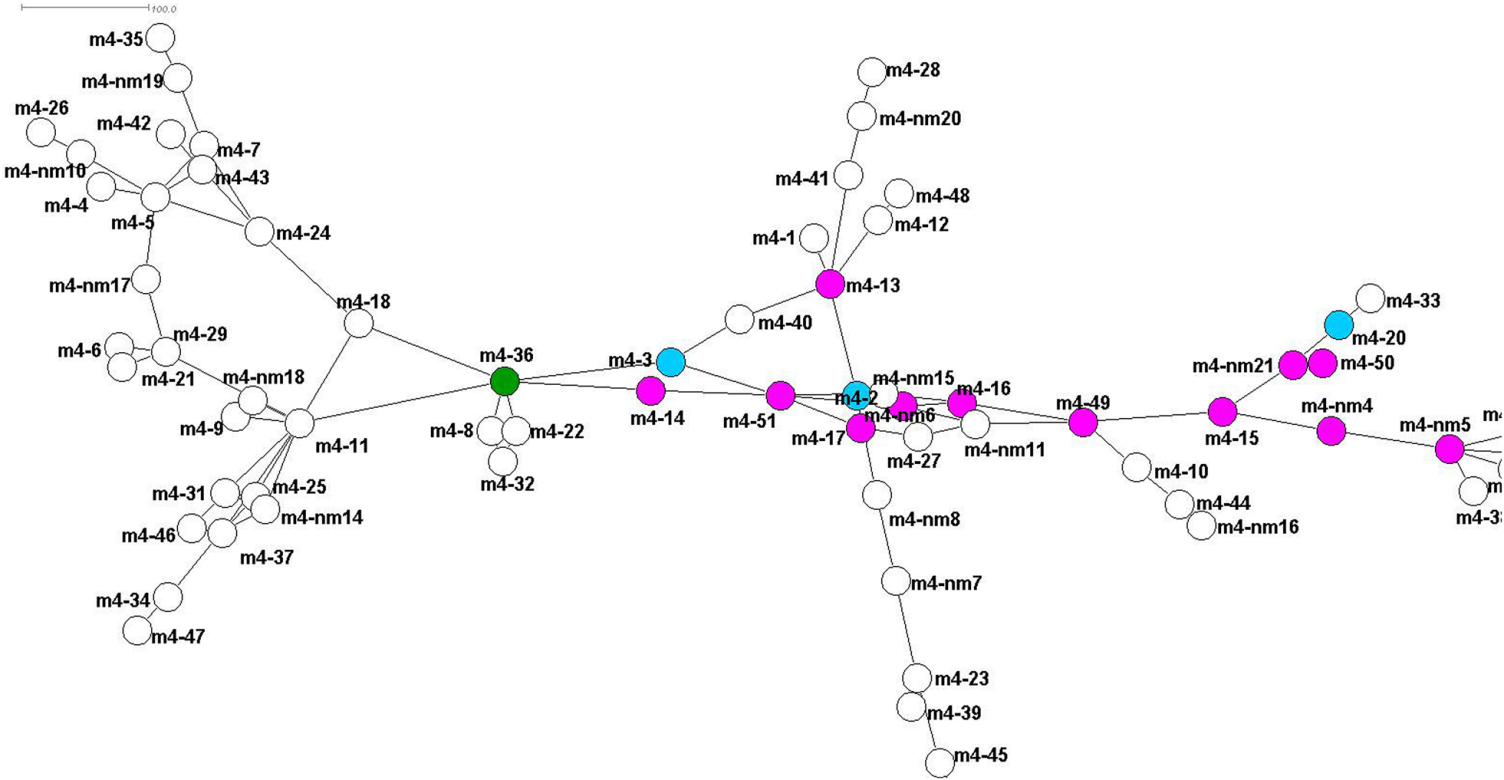
Minimum spanning net for distances on basis of a Kimura two-parameter (K2P) model corrected distance matrix from sequence alignment, showing the relations between the different *A. phagocytophilum msp4* variants determined from this study. The corresponding hosts and an exemplary sequence for each variant are given in Additional file 2: Table S6. Variants highlighted in pink include cattle as host, and those highlighted in blue include dogs as host; variant m4-36 connecting the two parts of the net is indicated in green. Labelling is explained in “Data compilation and naming of sequences” section

For *msp4*, 72 different variants from this study and from GenBank data (m4-nm) were analysed (Fig. 4; Additional file 2: Table S6). The net of the *msp4* gene exists in two distinct parts, connected by the variant m4-36 (marked green) which is derived from a fallow deer (Fig. 4). The left part of the net contains only variants from wildlife hosts (except for a variant found in small ruminants [m4-nm10] and a variant from a horse [m4-18]). The right part of the net shows a diverse mixture of variants found in wild and/or domestic animals; all cattle variants (marked with pink circles) are connected in the centre of this part of the net. In dogs, only three different variants are reported so far (marked blue).

**Fig. 5:**
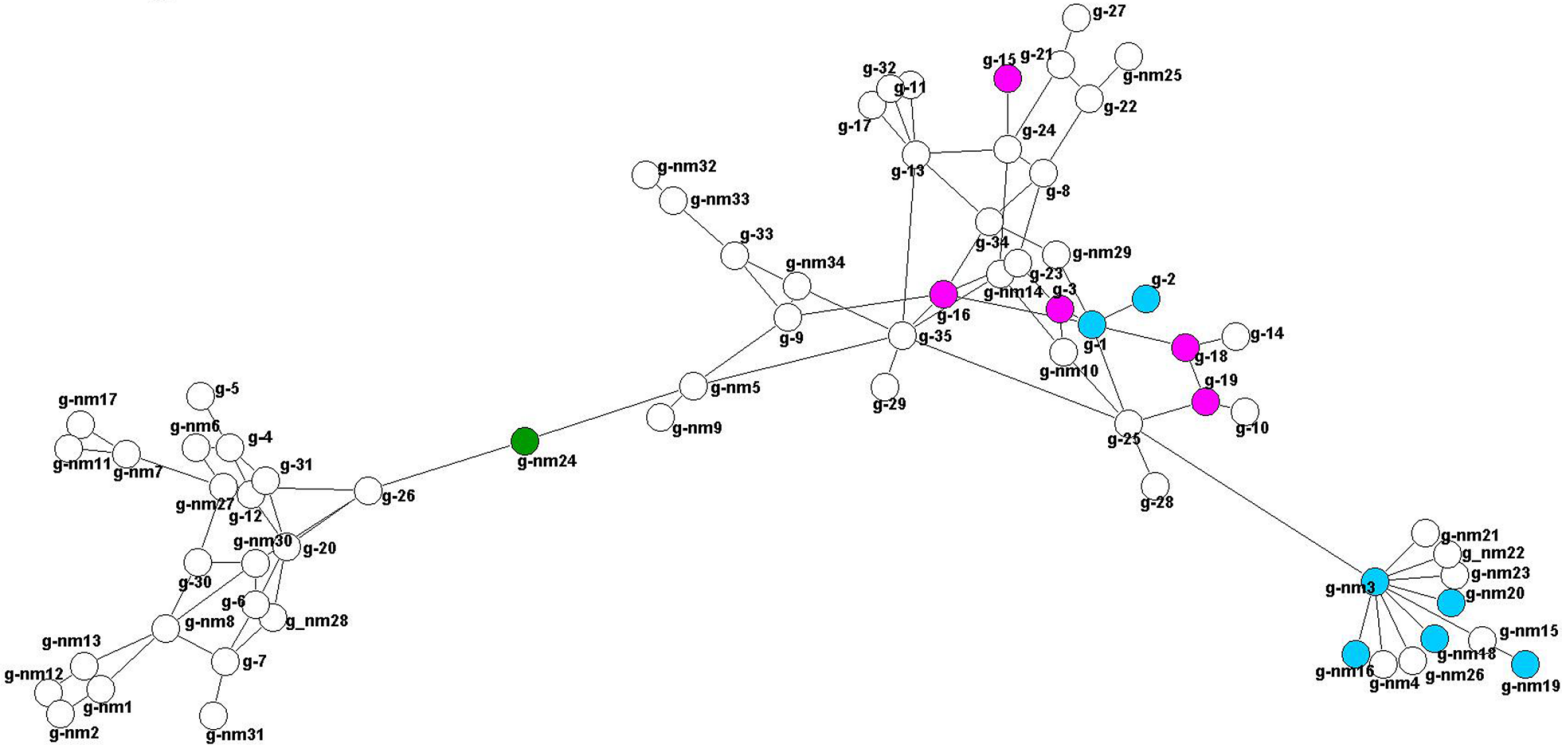
Minimum spanning net for distances on basis of a Kimura two-parameter (K2P) model corrected distance matrix from sequence alignment, showing the relations between the different *A. phagocytophilum groEL* variants determined from this study. The corresponding hosts and an exemplary sequence for each variant can be found in Additional file 2: Table S7. Variants highlighted in pink include cattle, and those highlighted in blue include dogs; the green circle shows variant g-nm24 connecting the two parts of the net. Labelling is explained in “Data compilation and naming of sequences” section

In total, 67 different *groEL* variants from this study and from GenBank were analysed (Additional file 2: Table S7). The net of the *groEL* gene has two distinct parts connected by the variant g-nm24 from an as-yet not further determined cervid sample (marked green) (Fig. 5). Variants associated with cattle, dog and domestic animals are found in the right part of the net exclusively, but those of different deer species and wildlife animals occur there as well. The left side of the net contains exclusively wild deer species (mostly roe deer) and rodents.

**Fig. 6:**
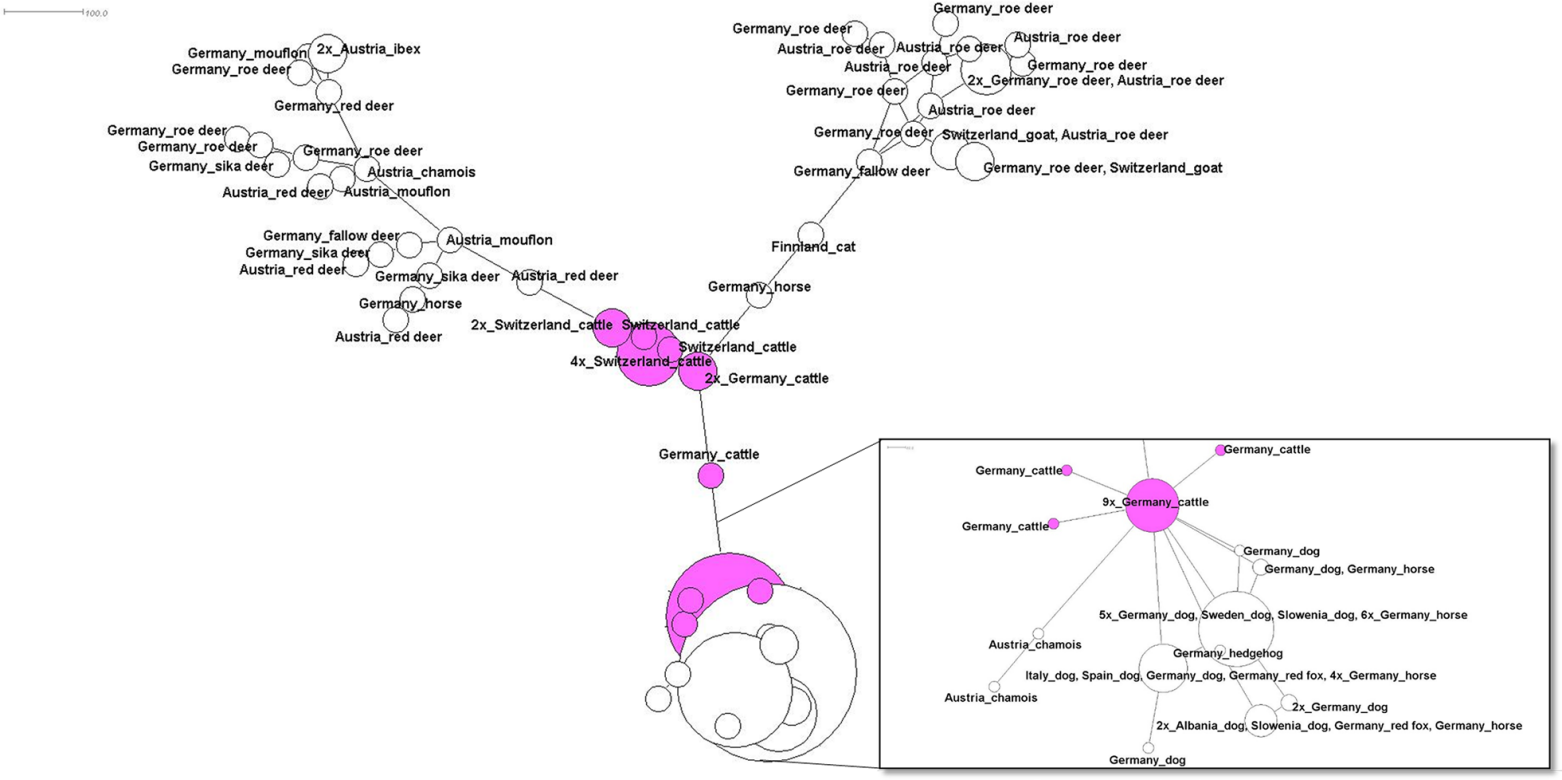
Minimum spanning net for distances on basis of a Kimura two-parameter (K2P) corrected distance matrix from sequence alignment, showing the relations between concatenated sequences of *16S* rRNA, *msp4* and *groEL* variants. Variants highlighted in pink are derived from cattle. *groEL*, Heat shock protein operon; *16S* rRNA, *16S* ribosomal RNA gene, *msp4 *major surface protein 4 gene

A concatenated dataset of 98 samples, including* 16S* rRNA,* msp4* and* groEL* sequences was generated. The resulting net is divided in three groups, of which two are associated with different wildlife species and the third contains dog, cattle and horse samples from different European countries (Fig. 6). The centre of the net is dominated by variants derived from cattle (pink).

## Discussion

To date, *A. phagocytophilum* has been detected in various wild and domestic hosts and a variety of different variants for several genetic markers was observed. The objective of this study was to assemble all individual results of genetic markers characterized in our group to expand current knowledge of variant-host associations for this pathogen. As stated earlier, modularity seems to be a key element in these constructs, and it characterizes the degree of interactions of variants from different gene loci, both among themselves but also with other genetic markers [6]. To unravel the complex circulation of *A. phagocytophilum* in Europe, we conducted multilocus analyses in combination with interactive network analyses to identify *A. phagocytophilum* lineages causing disease in ruminant and non-ruminant hosts [5].

### Suitability of the different gene loci to describe host–pathogen associations

The first aim of this study was to genotype and characterize samples of four partial genes (*16S* rRNA, *groEL*, *msp4*, *msp2*) of *A. phagocytophilum* from different mammalian host animals. The suitability of these gene loci for analysis of the host–pathogen interactions varies. Previous studies showed *msp2* to be a suitable marker for describing host–pathogen interaction [42]. Earlier studies also demonstrated that *msp2*, *p44* and other genes form a polymorphic multigene family encoding for major outer membrane proteins of *A. phagocytophilum*. The relative expression ratios of these genes seem to vary depending on the host [30], which might ease the evasion of specific immune responses of the host by antigenic variation [43] and complicate the interpretation of evolutionary divergence or spontaneous recombination on a single gene level [44]. Also, sequencing seems to be challenging as our analyses could only contribute 71 new sequences. These findings led us to decide not to further process this locus in the phylogenetic variant net analyses in the present study. In contrast, we found 174 new sequences with 50 different variants of *msp4*, thereby confirming the described high variability of this gene locus [18]. *Msp4* interacts with the immune system of vectors and hosts, potentially provoking faster evolution of new *A. phagocytophilum* strains due to higher selective pressure [45]. With the exception of variant m4-2(B/C), which was found in five different hosts, the *msp4* variants were clearly separated between ruminants and non-ruminants, confirming the results of a previous study by de la Fuente et al. who reported a different development of lineages specializing on ruminants and non-ruminants as hosts [46]. The most conserved gene locus in our analyses was *16S* rRNA, especially given the large number of sequences (*n* = 692). Although studies have distinguished between *16S* rRNA variants specific to red deer and those specific to roe deer [47], *16S* rRNA has been shown to not have enough discriminatory power to distinguish between distant lineages of *A. phagocytophilum* in Europe [5, 17, 23]. Additionally, our results confirmed that it is not sufficiently informative to use as a marker for evaluating the samples at specific host level, as equal variants were shared by diverse host species [9, 48]. For that reason, gene loci with a higher genetic variation of different nucleotide sequence patterns and therefore higher discriminatory power should be chosen [20]. Nevertheless, one of the most interesting variants is 16S-2(B), which was recently described to be a pathogenic variant for humans in the USA [14, 49] and also to infect various animal species, including dogs, horses, sheep, red deer and roe deer [9, 20, 50, 51]. This wide host range of the 16S-2(B) strain might reflect the high host adaptation skills of *A. phagocytophilum*. This was also confirmed in the present study in combination with variant m4-2(B/C), which occurred in several dogs and horses. This variant and variant combinations thereof might be an indicator for a broad host tropism, possibly supporting the conclusion that the complex interactions of hosts and vectors drive the evolutionary pressure and lead to new variants [6]. Our analyses also pointed out that the *groEL* gene locus is more diverse than the *16S* rRNA locus in terms of the number of occurring variants, but it is less diverse than *msp4* with regard to total number of variants. Variants g-1(A) and g-2(B) demonstrate a broad host tropism, with the majority of cases detected in domestic animals (Fig. 5). In addition, variant g-2(B) was associated with human infection in the past, detected in a Slovenian patient with a history of a tick bite [52]. The authors of a previous study revealed four distinct geographically dispersed ecotypes of the *groEL* locus, each with a significantly different host range in Europe [17]. On basis of these data, authors of earlier studies considered the *groEL* operon to be more suitable than the *16S* rRNA locus for distinguishing different geographic and pathogenetic variants of *A. phagocytophilum* [5, 53, 54].

### Evaluation of utilized methods and biases

All the results presented here are subject to a sampling bias because samples derived from hosts with obvious clinical symptoms and from wild animals represent convenience sampling, with a dependence on hunting regulations and season. The origin of many samples is Central Europe, but distribution is not equal throughout this area. In order to compensate for this sampling bias, we included additional *A. phagocytophilum* genes available from GenBank with diverse geographic origins in the analysis. The analyses of different partial genes in terms of genetic diversity is standard practice for the characterization of *A. phagocytophilum*. The current analyses support results and cycles that have been recently described and suggested. Nevertheless, extended analyses of a high number of genes or full genomes have been suggested recently [55, 56]. To achieve such analyses, a high number of host samples were tested by multilocus sequence typing, such as, for example, on goat samples [55], or a super tree was constructed on the basis of nine markers for French cattle variants [10]. Minimum spanning trees for cattle variants have also been produced recently [15]. Different analyses on single genes were conducted with the help of phylogenetic network-based methods to resolve host–vector interactions [6]. Referring to these previous studies, we have attempted to combine the multilocus approach (3 individual genes) with network analyses (minimum spanning nets for distances). We aimed at gaining additional information by these methods as data on variant analyses may not be tree-like since parallel evolution may occur due to the different evolutional cycles described for Europe. For this purpose, we combined net and multiple loci typing analyses. A minimum spanning net is easy to construct and far from being most parsimonious, but it is able to display sample sets differing in only a single nucleotide polymorphism (SNP) and it is easy to update with the advent of additional samples [57]. Also, we tried to overcome recent limitations of pattern density by concatenating three gene loci (*16S* rRNA, *msp4* and *groEL*); unfortunately the dataset with available sequences for all three gene loci was only available for 98 samples (Fig. 6). However, this result suggests a cattle-associated cycle and confirms previous studies on the importance of this host species [10, 15, 33]. For future perspectives, not only would analysis based on larger patterns or even full genomes be favourable, but the samples should additionally provide an extensively distributed origin, leading to new and additional insights into the endemic cycles and molecular epidemiology of this pathogen in Europe.

### Host selection of specific variants and pathogen life-cycle

As stated earlier, a high number of samples from this study originated from Germany, followed by other European countries and then by the USA. The existing spatial metadata are too heterogeneous for detailed phylogeographic analyses, and are neither balanced globally nor across continents. These limitations can be seen in the sampling bias, with overrepresentation of samples from Germany, and both hamper any possible interpretation of further geospatial analysis of variant-host connections and underline the need for more diverse sampling from different countries to fill this gap. The high number of investigated hosts prohibited individual evaluation of each host and, therefore, we focused our analyses on the hosts most commonly reported to be involved in the endemic life-cycles of *A. phagocytophilum*, namely dogs, due to their different clinical responses depending on the detected variant(s), and cattle, roe and red deer. For all of these hosts new sequences of different gene loci were created within the context of the present study, thus enhancing individual variant complexity and expanding current knowledge.

#### Dogs

In dogs, infections caused by *A. phagocytophilum* are mostly subclinical [58]. Nevertheless, it was previously hypothesized that different *16S* rRNA gene variants of *A. phagocytophilum* are involved in natural infections of dogs and that those might cause different clinical outcomes due to different levels of pathogenicity [32, 59]. In the present study, we predominantly sampled and further investigated samples from clinical cases, which may have introduced biased variant sampling. However, previous studies on canine blood donors revealed a 2.3% prevalence for *A. phagocytophilum* infection without clinical symptoms [60]. Figures 3 and 4 show seven different *16S* rRNA variants in dogs and only three *msp4* variants, which is contrary to the general variability of these gene loci. This finding may indicate the importance of considering *16S* rRNA analyses in the detection of *A. phagocytophilum* variants from dogs. Variant 16S-2(B) belongs to the inner nodes of the* 16S* rRNA spanning net, whereas variant 16S-1(A) is not connected to the inner nodes directly but to 16S-2(B) (Fig. 3); this result indicates that the 16S-1(A) variant is a more specific dog variant, where 16S-2(B) bridges the relation to all other variants. As ticks and hosts have been found with both of these variants, it is not possible to identify where the separation between 16S-1 (A) and 16S-2(B) took place. This result is also supported by Fig. 2, which shows that only two *msp4* variants are connected to cumulative different variant combinations of *16S* rRNA, *groEL* and *msp2*. As stated previously, these findings should be evaluated within the context of further studies, especially those focusing on the interactions of variants and pathogenicity [59]. Finally, it appears that dogs play a neglectable role in the endemic transmission cycles of *A. phagocytophilum*, which might only be interesting in urban areas [61], where its role needs to be delineated.

#### Cattle and deer and their role in life-cycles

Our results for cattle seem to indicate that cattle play a central role in the distribution and life-cycle of *A. phagocytophilum*, as visualized in the *msp4* net (Fig. 4) and in the concatenated net (Fig. 6). These figures demonstrate that there is a connection between all cattle variants (marked with pink circles on graphs) in the centre of both nets. The diversity of *16S* rRNA variants is limited in cattle samples [only two 16S-20(W) and one 16S-22(Y)], as presented in Fig. 2. The variant 16S-20(W) has been isolated from clinical cases, while 16S-22(Y) seems to be apathogenic [33, 62]. The 16S-20(W) variant also occurred in sheep with clinical abnormalities from Germany [63]. In general, this result may indicate a distinct host tropism for a number of *16S* rRNA *A. phagocytophilum* strains in cattle, while the diversity of other partial genes (*groEL*, *msp2* and *msp4*) is apparent. The role of cattle in the distribution and evolution of *A. phagocytophilum* has also been discussed for different geographical areas in earlier studies [15, 33, 64]. All of the observed cattle samples from this study originated from Germany or Switzerland. As stated earlier, 16S-20(W) and 16S-22(Y) were the main variants; only one isolate (GU236584) from GenBank showed variant 16S-21(X) (Fig. 2) and this sample with this variant originated from Norway [9]. For cattle samples with *msp4* from Germany, we found all variants, from m4-13(N) to m4-17(R)). Variant m4-14(O) also occurred in samples from a herd of cattle from Switzerland in combination with variant g-3 of the *groEL* operon, the 16S-20(W) and m2-3(b) for *msp2* variants [25]. Other *msp4* variants which were detected in German cattle are m4-49, m4-50 and m4-51 [33]. Variant g-15 and g-18 of the *groEL* operon as well as m2-26 for the *msp2* gene were detected in the new cattle sequences reported in the present study. For the GenBank sequences, we additionally detected m4-16 and m4-51 as well as three different “m4-nm” from French cattle. One of these French cattle variants, m4-nm5, is directly connected to the New World human Webster US strain, indicating a close relationship between both. In samples from Spanish cattle, we found one new “m4-nm” variant and variant m4-15(P) for the *groEL* operon; no additional variants were found, but we did identify a new msp2 (“m2-nm”) variant in cattle from Switzerland [46].

Unfortunately, only a limited number of red deer variant combinations were sequenced. Nevertheless, they all showed high individual variant combinations and some shared variants leading to different, but slightly overlapping transmission cycles of *A. phagocytophilum* [65]. As previously described, our red deer samples also contained the 16S-20 (W) variant which is common in cattle [5]. This is an interesting finding, which may be interpreted as being due to the different behaviour of red and roe deer: red deer seek closer contact to farm animals than roe deer. This finding supports the assumption that red deer may play a more important role as potential reservoir hosts for domestic ruminant strains than roe deer [21]. It has been experimentally shown that red deer act as reservoir for *A. phagocytophilum* strains pathogenic for sheep [66]. In addition, variants detected in red deer have been described to be pathogenic for humans, dogs, horses and domestic ruminants [20, 67, 68]. In contrast, roe deer seem to be only sporadically involved in the circulation of the pathogenic strains of *A. phagocytophilum 16S* rRNA or *groEL* variants [67]. In general, there is a high prevalence for *A. phagocytophilum* in roe deer, up to 98.9%, indicating the susceptibility of this species to this pathogen [26]. Our analyses showed that roe deer had the highest number of variants for all four genes, which could be related to intense roaming activities of this species, resulting in increasing contact with different vectors and hosts and therefore with different variants. All of these gene variants are also diversly connected to one another in different hosts, without any clear variant-combination system (Fig. 2). This diversity was described already earlier for *groEL* variants [47, 69, 70]. Most of the *groEL* variants circulate between ticks and roe deer exclusively, and only a few have been reported to be transmitted to domestic animals and humans as dead end hosts [23]. The occurrence of an individual roe deer subcycle was proposed earlier based on different investigated genes [5, 17, 18, 71]. Additionally, this species is considered to be a reservoir host in both suggested life-cycles of *A. phagocytophilum* in Europe.

In general, our data confirm both hypothesized life-cycles of *A. phagocytophilum* in Europe. The first of these life-cycles includes hedgehogs, red foxes and red deer, among others, as potential reservoir hosts and domestic animals and humans as potential hosts that develop clinical symptoms of the disease. The lack of a sufficient number of samples from red deer has unfortunately hampered analyses of the reservoir host theory [67] for this species, and our study has not provided new insight on whether questioning of this hypotheses is justified [10, 21]. Hedgehogs as possible reservoir hosts were confirmed by the present study. The samples for hedgehogs showed high uniformity of *A. phagocytophilum* strains that also occur in domestic animals and humans and, therefore, their role as reservoir host is supported [24, 72]. For the* msp4* and *groEL* variants (Figs. 4, 5), a two-parted net can be seen. The right side of the net supports previously suggested life-cycles [5, 33], and the left side of the net also suggests a deer–small ruminant (sheep, goat)–rodent–mouflon cycle that is completely separated from that of the dog or cattle variants. This additional cycle could also explain the individual, unconnected clusters of roe deer variant combinations in Fig. 2.

The second endemic cycle of *A. phagocytophilum* possibly involves wild cervids and rodents as reservoir hosts and domestic ruminants as hosts [5]. Unfortunately we could not add new sequence variants of *A. phagocytophilum* for rodents, but our analyses suggested a more diverse role of rodents in the life-cycle of *A. phagocytophilum* as proposed in previous studies. Authors of earlier studies concluded that rodents are unlikely reservoir hosts for *A. phagocytophilum* from domestic animals and humans [17, 48]. Conversely, in the present study, the minimum spanning nets of *16S* rRNA (Fig. 3) and *groEL* (Fig. 5) show rodent variants (16Snm5 16Snm24, 16Snm25, gnm15, gnm4, gnm3) closely related to those of cat, dog, horse and human. For *groEL*, all of these variants are distant to the cattle, dog and deer subnet, suggesting an additional subcycle. These findings need to be clarified by further analyses of additional rodent samples.

## Conclusion

To unravel the complex transmission cycles of tick-borne pathogens such as *A. phagocytophilum* and to understand variant combinations in different host species, we used a complex toolset for molecular epidemiology, phylogeny and network theory. Interestingly, different hosts showed unique but also shared variant combinations and variant distributions. Cattle seem to play a central role in the distribution of variants. The present study included nearly 1300 sequences and confirmed and extended current knowledge on the two previously hypothesized life-cycles of this pathogen in Europe. This information could be used to set the basis to develop European spread-and-infection risk analyses for *A. phagocytophilum*. The use of three (partly four) individual gene loci already expand current knowledge of host–pathogen interactions on the basis of insights in individual variability of different gene loci from different hosts.

### Supplementary Information

**Additional file 1: Table S1.** Accession numbers of all sequences included in any analyses within this study.

**Additional file 2: Table S2.** Primer and probes for *16S* rRNA, *msp4*, *msp2* and *groEL*. **Table S3.** Samples with 4/4, 3/4 and 2/4 partial gene variants. **Table S4.** Distribution of the* msp2* consensus variants in sequences (sequences derived in group’ own study and from GenBank). **Table S5.** Distribution of the *16S* rRNA variants in the different animal species (sequences derived in group’s own study and from GenBank). **Table S6.** Distribution of the *msp4* variants in different animal species (sequences obtained from group’s own study and GenBank. **Table S7.** Distribution of the *groEL* variants in the different animal species (sequences derived in group’s own study and from GenBank).

## Data Availability

The datasets supporting the conclusions of this article are included within the article (and its additional files).

## Abbreviations

*groEL*: Heat shock operon
K2P: Kimura-2-parameter model
MLST: Multilocus sequence typing
*msp2*: Major surface protein 2 gene
*msp4*: Major surface protein 4 gene
PCR: Polymerase chain reaction
SNP: Single-nucleotide polymorphism
*16S* rRNA: *16S* ribosomal RNA
