# Supplementary material for: Host–pathogen associations revealed by genotyping of European strains of Anaplasma phagocytophilum to describe natural endemic cycles
Source: Parasit Vectors. 2023 Aug 16;16:289. doi: 10.1186/s13071-023-05900-3 (PMC10433637; doi:10.1186/s13071-023-05900-3)
Supplement: Supplementary file 2 — Additional file 2: Table S2. Primer and probes for 16S rRNA, msp4, msp2 and groEL. Table S3. Samples with 4/4, 3/4 and 2/4 partial gene variants. Table S4. Distribution of the msp2 consensus variants in sequences (sequences derived in group’ own study and from GenBank). Table S5. Distribution of the 16S rRNA variants in the different animal species (sequences derived in group’s own study and from GenBank). Table S6. Distribution of the msp4 variants in different animal species (sequences obtained from group’s own study and GenBank. Table S7. Distribution of the groEL variants in the different animal species (sequences derived in group’s own study and from GenBank). [file 13071_2023_5900_MOESM2_ESM.docx]

**Table S2: Primer and Probes for *16S* rRNA, *msp4*, *msp2* and *groEL***

| **Target Gene** | **Primer 5'-3'** | **Cycle conditions** | **Reference** |
| --- | --- | --- | --- |
| *16S* rRNA | **1st amplification**  *ge3a* CACATGCAAGTCGAACGGATTATTC  *ge10r* TTCCGTTAAGAAGGATCTAATCTCC  **2nd amplification**  *ge9f* AACGGATTATTCTTTATAGCTTGCT  *ge2* GGCAGTATTAAAAGCAGCTCCAGG | 40 cycles (2^nd^ 25 cycles)  30 sec 94°C, 30 sec 55°C, 60 sec 72°C | [1] |
| *msp2* | *ApMSP2f* ATGGAAGGTAGTGTTGGTTATGGTATT  *ApMSP2*r TTGGTCTTGAAGCGCTCGTA  *ApMSP2p* TGGTGCCAGGGTTGAGCTTGAGATTG  labeled 5'-FAM, 3'-TAMRA  *msp25* TTATGATTAGGCCTTTGGGCATG  *msp23* TCAGAAAGATACACGTGCGCCC | 50 cycles  15 sec 94°C, 60 sec 60°C  35 cycles  60 sec 95°C, 60 sec 62°C, 90 sec 72°C | [2]  [3] |
| *msp4* | **1st amplification**  *Msp4AP5* ATGAATTACAGAGAATTGCTTGTAGG  *Msp4AP3* TTAATTGAAAGCAAATCTTGCTCCTATG  **2nd amplification**  *Msp4f* CTATTGGYGGNGCYAGAGT  *Msp4r* GTTCATCGAAAATTCCGTGGTA | 40 cycles  30 sec 94°C, 45 sec 54°C, 60 sec 72°C | [4]  [5] |
| *groEL* | **1st amplification**  *AphplgroELF* ATGGTATGCAGTTTGATCGC  *AphplgroELR* TCTACTCTGTCTTTGCGTTC  **2nd amplification**  *AphplgroELF* ATGGTATGCAGTTTGATCGC  *EphgroELR* TTGAGTACAGCAACACCACCGGAA | 40 cycles  30 sec 94°C, 30 sec 55°C, 45 sec 72°C | [6, 7] |

**Table S3: Samples with 4/4, 3/4 and 2/4 partial gene variants**

| **Sample ID** | **Animal**  **species** | **Origin** |  | **partial gens** |  |  |
| --- | --- | --- | --- | --- | --- | --- |
|  |  |  | ***16S* rRNA** | ***msp2*** | ***msp4*** | ***groEL*** |
| 14333 | Dog | Slovenia | 16S-1(A) | m2-13 | m4-20 | g-1(A) |
| 8074 | Dog | Slovenia | 16S-2(B) | m2-13 | m4-2(B/C) | g-2(B) |
| BwReh9 | Roe deer | Germany | 16S-20(W) | m2-17 | m4-42 | g-24 |
| BwReh12 | Roe deer | Germany | 16S-22(Y) | m2-19 | m4-13(N) | g-7(G) |
| 9774 | Dog | Spain | 16S-2(B) | m2-2(a) | m4-2(B/C) | g-2(B) |
| 633200.1 | Dog | Germany | 16S-2(B) | m2-2(a) | m4-2(B/C) | g-2(B) |
| 630300 | Dog | Germany | 16S-2(B) | m2-2(a) | m4-2(B/C) | g-2(B) |
| 6380 | Dog | Germany | 16S-2(B) | m2-2(a) | m4-2(B/C) | g-2(B) |
| 5937 | Dog | Germany | 16S-2(B) | m2-2(a) | m4-2(B/C) | g-2(B) |
| 7641 | Dog | Germany | 16S-2(B) | m2-2(a) | m4-2(B/C) | g-2(B) |
| S 1710/05 | Dog | Sweden | 16S-2(B) | m2-2(a) | m4-2(B/C) | g-2(B) |
| 50.Bella | Dog | Germany | 16S-2(B) | m2-2(a) | m4-2(B/C) | g-2(B) |
| S 654/04 | Horse | Germany | 16S-2(B) | m2-2(a) | m4-2(B/C) | g-2(B) |
| S 1829/04 | Horse | Germany | 16S-2(B) | m2-2(a) | m4-2(B/C) | g-2(B) |
| S 1741/07 | Horse | Germany | 16S-2(B) | m2-2(a) | m4-2(B/C) | g-2(B) |
| S 2614/07 | Horse | Germany | 16S-2(B) | m2-2(a) | m4-2(B/C) | g-2(B) |
| M8 Okt | Goat | Switzerland | 16S-21(X) | m2-2(a) | m4-1(A) | g-5(E) |
| BwReh13 | Roe deer | Germany | 16S-21(X) | m2-20 | m4-13(N) | g-30 |
| BwReh14 | Roe deer | Germany | 16S-21(X) | m2-21 | m4-13(N) | g-5(E) |
| D31 | Roe deer | Germany | 16S-19(V) | m2-22 | m4-35 | g-4(D) |
| 7_19.06. | Cattle | Germany | 16S-20(W) | m2-26 | m4-49 | g-18(X) |
| 14_04.06. | Cattle | Germany | 16S-20(W) | m2-26 | m4-49 | g-18(X) |
| 22_30.05. | Cattle | Germany | 16S-20(W) | m2-26 | m4-49 | g-18(X) |
| 46_22.05. | Cattle | Germany | 16S-20(W) | m2-26 | m4-49 | g-18(X) |
| 57_04.06. | Cattle | Germany | 16S-20(W) | m2-26 | m4-49 | g-18(X) |
| 61_22.06. | Cattle | Germany | 16S-20(W) | m2-26 | m4-49 | g-18(X) |
| 58_17.05. | Cattle | Germany | 16S-20(W) | m2-26 | m4-50 | g-18(X) |
| 11167 | Cattle | Switzerland | 16S-20(W) | m2-3(b) | m4-14(O) | g-3(C) |
| 11047 | Cattle | Switzerland | 16S-20(W) | m2-3(b) | m4-14(O) | g-3(C) |
| 11168 | Cattle | Switzerland | 16S-20(W) | m2-3(b) | m4-14(O) | g-3(C) |
| 11023 | Cattle | Switzerland | 16S-20(W) | m2-3(b) | m4-14(O) | g-3(C) |
| 11208 | Cattle | Switzerland | 16S-20(W) | m2-3(b) | m4-14(O) | g-3(C) |
| 11078 | Cattle | Switzerland | 16S-20(W) | m2-3(b) | m4-14(O) | g-3(C) |
| 11046 | Cattle | Switzerland | 16S-20(W) | m2-3(b) | m4-14(O) | g-3(C) |
| 10946 | Cattle | Switzerland | 16S-20(W) | m2-3(b) | m4-14(O) | g-3(C) |
| 11516 | Cattle | Switzerland | 16S-20(W) | m2-3(b) | m4-14(O) | g-3(C) |
| S 1201/05 | Horse | Germany | 16S-3(D) | m2-4(d) | m4-3(D) | g-13(L) |
| 6933 | Dog | Germany | 16S-1(A) | m2-5(e) | m4-2(B/C) | g-1(A) |
| 7444 | Dog | Italy | 16S-2(B) | m2-5(e) | m4-20 | g-2(B) |
| S 2630/07 | Horse | Germany | 16S-2(B) | m2-5(e) | m4-2(B/C) | g-2(B) |
| S 1071/08 | Horse | Germany | 16S-2(B) | m2-5(e) | m4-2(B/C) | g-2(B) |
| S 1220/08 | Horse | Germany | 16S-2(B) | m2-5(e) | m4-2(B/C) | g-2(B) |
| S 1379/06 | Horse | Germany | 16S-2(B) | m2-5(e) | m4-2(B/C) | g-2(B) |
| S1074/09 | Horse | Germany | 16S-2(B) | m2-5(e) | m4-2(B/C) | g-2(B) |
| S1025/09 | Horse | Germany | 16S-2(B) | m2-5(e) | m4-2(B/C) | g-2(B) |
| S1085/09 | Horse | Germany | 16S-2(B) | m2-5(e) | m4-2(B/C) | g-2(B) |
| S146 | Red fox | Germany | 16S-1(A) | m2-6(f) | m4-20 | g-1(A) |
| 80 | Hedgehog | Germany | 16S-1(A) | m2-6(f) | m4-20 | g-1(A) |
| S 1729/08 | Horse | Germany | 16S-1(A) | m2-6(f) | m4-2(B/C) | g-1(A) |
| 680200 | Dog | Germany | 16S-1(A) | m2-6(f) | m4-2(B/C) | g-1(A) |
| 971 | Cat | Finland | 16S-13(O) | m2-6(f) | m4-2(B/C) | g-2(B) |
| S 1523/07 | Horse | Germany | 16S-16(S) | m2-6(f) | m4-18(S) | g-13(L) |
| BwSi3 | Sika deer | Germany | 16S-16(S) |  | m4-31 | g-34 |
| BwSi12 | Sika deer | Germany | 16S-20(W) |  | m4-32 | g-13(L) |
| M19 | Fallow deer | Germany | 16S-2(B) |  | m4-36 | g-8(H) |
| Rotwild23 | Red deer | Austria | 16S-28 |  | m4-38 | g-13(L) |
| BwReh16 | Roe deer | Germany | 16S-7(I) |  | m4-39 | g-7(G) |
| Rothirsch3 | Red deer | Austria | 16S-8(J) |  | m4-4(J) | g-8(H) |
| M20 | Fallow deer | Germany | 16S-22(Y) |  | m4-40 | g-29 |
| Gams16 | Chamois | Austria | 16S-20(W) |  | m4-10(K) | g-10(J) |
| Gams15 | Chamois | Austria | 16S-20(W) |  | m4-10(K) | g-9(I) |
| Reh19 | Roe deer | Austria | 16S-21(X) |  | m4-12(M) | g-12(K) |
| Reh26 | Roe deer | Austria | 16S-16(S) |  | m4-13(N) | g-7(G) |
| BwReh10 | Roe deer | Germany | 16S-21(X) |  | m4-13(N) | g-20 |
| Reh24 | Roe deer | Austria | 16S-21(X) |  | m4-13(N) | g-6(F) |
| Steinbock6 | Ibex | Austria | 16S-16(S) |  | m4-13(N) | g-6(F) |
| Reh20 | Roe deer | Austria | 16S-21(X) |  | m4-13(N) | g-7(G) |
| Steinbock5 | Ibex | Austria | 16S-16(S) |  | m4-13(N) | g-6(F) |
| BwReh24 | Roe deer | Germany | 16S-22(Y) |  | m4-13(N) | g-31 |
| Reh23 | Roe deer | Austria | 16S-22(Y) |  | m4-13(N) | g-6(F) |
| Reh27 | Roe deer | Austria | 16S-22(Y) |  | m4-13(N) | g-7(G) |
| Reh25 | Roe deer | Austria | 16S-22(Y) |  | m4-13(N) | g-7(G) |
| BwReh8 | Roe deer | Germany | 16S-22(Y) |  | m4-13(N) | g-7(G) |
| A31 | Mouflon | Germany | 16S-21(X) |  | m4-21 | g-21 |
| 69 | Dog | Albania | 16S-1(A) |  | m4-20 | g-1(A) |
| 438 | Dog | Albania | 16S-1(A) |  | m4-20 | g-1(A) |
| S1360/09 | Dog | Germany | 16S-2(B) |  | m4-20 | g-2(B) |
| 150580 | Dog | Germany | 16S-2(B) |  | m4-2(B/C) | g-2(B) |
| 153681 | Dog | Germany | 16S-2(B) |  | m4-20 | g-2(B) |
| BwRo4 | Red deer | Germany | 16S-20(W) |  | m4-23 | g-33 |
| K41 | Roe deer | Germany | 16S-20(W) |  | m4-45 | g-25 |
| BwSi11 | Sika deer | Germany | 16S-29 |  | m4-45 | g-28 |
| S226 | Red fox | Germany | 16S-2(B) |  | m4-20 | g-2(B) |
| Z 31-27 | Cattle | Germany | 16S-20(W) |  | m4-15(P) | g-16(O) |
| Z 32-4 | Cattle | Germany | 16S-20(W) |  | m4-16(Q) | g-19(Y) |
| Z 32-6 | Cattle | Germany | 16S-20(W) |  | m4-17(R) | g-18(X) |
| 89 | Hedgehog | Germany | 16S-20(W) |  | m4-20 | g-1(A) |
| L12 | Roe deer | Germany | 16S-22(Y) |  | m4-48 | g-7(G) |
| 53_22.06. | Cattle | Germany | 16S-20(W) |  | m4-49 | g-18(X) |
| Gams25 | Chamois | Austria | 16S-20(W) |  | m4-5(I) | g-16(O) |
| Gams24 | Chamois | Austria | 16S-20(W) |  | m4-5(I) | g-8(H) |
| Mufflon3 | Mouflon | Austria | 16S-20(W) |  | m4-5(I) | g-8(H) |
| Mufflon4 | Mouflon | Austria | 16S-22(Y) |  | m4-5(I) | g-8(H) |
| 22_03.10. | Cattle | Germany | 16S-20(W) |  | m4-51 | g-15(N) |
| 28_3.10. | Cattle | Germany | 16S-20(W) |  | m4-51 | g-15(N) |
| 49_19.05. | Cattle | Germany | 16S-20(W) |  | m4-51 | g-15(N) |
| BwRo2 | Red deer | Germany | 16S-20(W) |  | m4-7(F) | g-32 |
| Rothirsch14 | Red deer | Austria | 16S-16(S) |  | m4-8(H) | g-7(G) |
| Rotwild2 | Red deer | Austria | 16S-17(T) |  | m4-9(G) | g-6(F) |
| B11 Juni | Goat | Switzerland | 16S-22(Y) |  | m4-1(A) | g-4(D) |
| 1 | Hedgehog | Germany | 16S-1(A) |  |  | g-1(A) |
| 20 | Hedgehog | Germany | 16S-1(A) |  |  | g-1(A) |
| 79 | Hedgehog | Germany | 16S-1(A) |  |  | g-1(A) |
| 98 | Hedgehog | Germany | 16S-1(A) |  |  | g-1(A) |
| 107 | Hedgehog | Germany | 16S-1(A) |  |  | g-1(A) |
| 126 | Hedgehog | Germany | 16S-1(A) |  |  | g-1(A) |
| 151 | Hedgehog | Germany | 16S-1(A) |  |  | g-1(A) |
| 153 | Hedgehog | Germany | 16S-1(A) |  |  | g-1(A) |
| 154 | Hedgehog | Germany | 16S-1(A) |  |  | g-1(A) |
| 160 | Hedgehog | Germany | 16S-1(A) |  |  | g-1(A) |
| 197 | Hedgehog | Germany | 16S-1(A) |  |  | g-1(A) |
| 261 | Hedgehog | Germany | 16S-1(A) |  |  | g-1(A) |
| 262 | Hedgehog | Germany | 16S-1(A) |  |  | g-1(A) |
| 7225 | Dog | Spain | 16S-1(A) |  |  |  |
| 7996 | Dog | Germany | 16S-1(A) |  | m4-2(B/C) |  |
| S 2070/03 | Dog | Germany | 16S-1(A) | m2-6(f) |  |  |
| 155480 | Dog | Germany | 16S-1(A) |  |  | g-1(A) |
| 8210 | Dog | Germany | 16S-1(A) |  |  | g-1(A) |
| S133 | Red fox | Germany | 16S-1(A) |  |  | g-1(A) |
| 413 | Dog | Albania | 16S-1(A) |  |  | g-1(A) |
| Rotwild22 | Red deer | Austria | 16S-2(B) |  | m4-37 |  |
| BwSi23 | Sika der | Germany | 16S-2(B) |  | m4-21 |  |
| 144881 | Dog | Germany | 16S-2(B) |  |  | g-2(B) |
| 8340 | Dog | Germany | 16S-2(B) |  |  | g-2(B) |
| Nike | Dog | Switzerland | 16S-2(B) |  | m4-20 |  |
| B17 | Mouflon | Germany | 16S-2(B) |  | m4-22 |  |
| K26 | Mouflon | Germany | 16S-2(B) |  |  | g-13(L) |
| BwReh6 | Roe deer | Germany | 16S-7(I) |  | m4-23 |  |
| BwReh31 | Roe deer | Germany | 16S-7(I) |  | m4-23 |  |
| BwReh33 | Roe deer | Germany | 16S-7(I) |  |  | g-7(G) |
| BwSi14 | Sika deer | Germany | 16S-8(J) |  | m4-33 |  |
| 150516 | Dog | Germany | 16S-9(K) |  | m4-2(B/C) |  |
| Rothirsch6 | Red deer | Austria | 16S-16(S) |  |  | g-7(G) |
| Mufflon12 | Mouflon | Austria | 16S-16(S) |  |  | g-21 |
| BwSi7 | Sika deer | Germany | 16S-16(S) |  | m4-44 |  |
| BwRo1 | Red deer | Germany | 16S-16(S) |  | m4-43 |  |
| BwRo5 | Red deer | Germany | 16S-16(S) |  | m4-25 |  |
| BwSi13 | Sika deer | Germany | 16S-16(S) |  | m4-46 |  |
| BwSi19 | Sika deer | Germany | 16S-16(S) |  | m4-34 |  |
| BwRo14 | Red deer | Germany | 16S-16(S) |  | m4-28 |  |
| H24 | Mouflon | Germany | 16S-16(S) |  | m4-5(I) |  |
| K37 | Mouflon | Germany | 16S-16(S) |  |  | g-8(H) |
| Gams38 | Chamois | Austria | 16S-20(W) |  |  | g-27 |
| BwRo6 | Red deer | Germany | 16S-20(W) |  | m4-26 |  |
| BwDa3 | Fallow deer | Germany | 16S-20(W) |  |  | g-24 |
| BwRo15 | Red deer | Germany | 16S-20(W) |  | m4-29 |  |
| BwRo18 | Red deer | Germany | 16S-20(W) |  | m4-30 |  |
| E09 | Mouflon | Germany | 16S-20(W) |  | m4-8(H) |  |
| L35 | Mouflon | Germany | 16S-20(W) |  | m4-5(I) |  |
| L38 | Mouflon | Germany | 16S-20(W) |  |  | g-13(L) |
| N04 | Mouflon | Germany | 16S-20(W) |  |  | g-34 |
| O32 | Mouflon | Germany | 16S-20(W) |  |  | g-23 |
| 22_09.07. | Cattle | Germany | 16S-20(W) |  | m4-50 |  |
| 42_04.09. | Cattle | Germany | 16S-20(W) |  | m4-49 |  |
| 57_04.09. | Cattle | Germany | 16S-20(W) |  | m4-49 |  |
| BwSi17 | Sika deer | Germany | 16S-21(X) |  | m4-47 |  |
| BwReh32 | Roe deer | Germany | 16S-21(X) |  |  | g-6(F) |
| B10 Juni | Goat | Switzerland | 16S-21(X) |  | m4-2(B/C) |  |
| E25 | Roe deer | Germany | 16S-21(X) |  | m4-41 |  |
| O08 | Roe deer | Germany | 16S-21(X) |  |  | g-26 |
| BwReh15 | Roe deer | Germany | 16S-22(Y) |  | m4-12(M) |  |
| A04 | Roe deer | Germany | 16S-22(Y) |  |  | g-7(G) |
| P10 | Roe deer | Germany | 16S-22(Y) |  |  | g-20 |
| 59_04.09. | Cattle | Germany | 16S-22(Y) |  | m4-13(N) |  |
| A8 Juli | Goat | Switzerland | 16S-23(Z) |  | m4-2(B/C) |  |
| S220 | Red fox | Germany | 16S-27 |  | m4-20 |  |
| BwRo3 | Red deer | Germany | 16S-30 |  | m4-24 |  |
| BwReh4 | Roe deer | Germany |  |  | m4-2(B/C) | g-6(F) |
| 212 | Hedgehog | Germany |  |  | m4-20 | g-1(A) |
| 61_09.07. | Cattle | Germany |  | m2-27 | m4-51 |  |
|  |  |  |  |  |  |  |
|  |  |  |  |  |  |  |

**Table S4:** **Distribution of the *msp2* consensus variants in sequences (sequences derived in own study and from GenBank)**

| **Host**  **Variant**  **exemplary Accession** | **Human** | **Dog** | **Horse** | **Bear** | **Cat** | **Hedgehog** | **Red fox** | **Cattle** | **Sheep** | **Goat** | **Roe deer** | **Red deer** | **Fallow deer** | **Rodent** | **Total** |
| --- | --- | --- | --- | --- | --- | --- | --- | --- | --- | --- | --- | --- | --- | --- | --- |
| **m2-2(a)**  **JN656318** |  | 11 | 4 |  |  |  |  |  |  | 1 |  |  |  |  | 16 |
| **m2-3(b)**  **OQ366413** |  |  |  |  |  |  |  | 9 |  |  |  |  |  |  | 9 |
| **m2-4(d)**  **JF893903** |  |  | 1 |  |  |  |  |  |  |  |  |  |  |  | 1 |
| **m2-5(e)**  **JF893904** |  | 2 | 7 |  |  |  |  |  |  |  |  |  |  |  | 9 |
| **m2-6(f)**  **KX395921** |  | 3 | 2 |  | 1 | 1 | 1 |  |  |  |  |  |  |  | 8 |
| **m2-11(n)**  **FJ812385** |  |  |  |  |  |  |  |  |  |  |  | 1 |  |  | 1 |
| **m2-12(p)**  **FJ812384** |  |  |  |  |  |  |  |  |  |  |  | 1 |  |  | 1 |
| **m2-13**  **KX395924** |  | 2 |  |  |  |  |  |  |  |  |  |  |  |  | 2 |
| **m2-14**  **KX395926** |  | 1 |  |  |  |  |  |  |  |  |  |  |  |  | 1 |
| **m2-15**  **KX395929** |  |  |  |  |  | 1 |  |  |  |  |  |  |  |  | 1 |
| **m2-16**  **KX395928** |  |  |  |  |  | 1 |  |  |  |  |  |  |  |  | 1 |
| **m2-17**  **OQ366422** |  |  |  |  |  |  |  |  |  |  | 1 |  |  |  | 1 |
| **m2-18**  **KX448795** |  |  |  |  |  |  |  |  |  |  |  | 1 |  |  | 1 |
| **m2-19**  **KX448794** |  |  |  |  |  |  |  |  |  |  | 1 |  |  |  | 1 |
| **m2-20**  **KX448793** |  |  |  |  |  |  |  |  |  |  | 1 |  |  |  | 1 |
| **m2-21**  **KX395927** |  |  |  |  |  |  |  |  |  |  | 1 |  |  |  | 1 |
| **m2-22**  **KX448792** |  |  |  |  |  |  |  |  |  |  | 1 |  |  |  | 1 |
| **m2-23**  **KX448791** |  |  |  |  |  |  |  |  |  |  | 1 |  |  |  | 1 |
| **m2-24**  **KX448796** |  |  |  |  |  |  |  |  |  |  |  |  | 1 |  | 1 |
| **m2-25**  **KX448790** |  |  |  |  |  | 1 |  |  |  |  |  |  |  |  | 1 |
| **m2-26**  **KU587064** |  |  |  |  |  |  |  | 9 |  |  |  |  |  |  | 9 |
| **m2-27**  **KU587067** |  |  |  |  |  |  |  | 2 |  |  |  |  |  |  | 2 |
| **m2-nm1**  **AY541006** | 2 |  |  |  |  |  |  |  |  |  |  |  |  |  | 2 |
| **m2-nm2**  **FJ467336** |  |  | 1 |  |  |  |  |  |  |  |  |  |  |  | 1 |
| **m2-nm3**  **FJ467335** |  | 1 |  |  |  |  |  |  |  |  |  |  |  | 3 | 1 |
| **m2-nm4**  **FJ467334** |  | 1 |  |  |  |  |  |  |  |  |  |  |  |  | 1 |
| **m2-nm5**  **FJ467333** |  | 1 |  |  |  |  |  |  |  |  |  |  |  |  | 1 |
| **m2-nm6**  **AY164494** | 1 |  |  |  |  |  |  |  |  |  |  |  |  |  | 1 |
| **m2-nm7**  **AY164493** | 1 |  |  |  |  |  |  |  |  |  |  |  |  |  | 1 |
| **m2-nm8**  **AY164492** | 1 |  |  |  |  |  |  |  |  |  |  |  |  |  | 1 |
| **m2-nm9**  **AY164491** | 1 |  |  |  |  |  |  |  |  |  |  |  |  |  | 1 |
| **m2-nm10**  **AY164490** | 1 |  |  |  |  |  |  |  |  |  |  |  |  |  | 1 |
| **m2-nm11**  **AY706393** |  |  |  |  |  |  |  |  | 1 |  |  |  |  |  | 1 |
| **m2-nm12**  **AY706392** |  |  |  |  |  |  |  | 1 |  |  |  |  |  |  | 1 |
| **m2-nm13**  **AY541005** | 1 |  |  |  |  |  |  |  |  |  |  |  |  |  | 1 |
| **m2-nm14**  **AY541004** |  |  |  |  |  |  |  |  | 1 |  |  |  |  |  | 1 |
| **m2-nm15**  **DQ519570** |  |  |  |  |  |  |  |  |  |  |  |  |  | 1 | 1 |
| **m2-nm16**  **DQ519569** |  |  |  |  |  |  |  |  | 1 |  |  |  |  |  | 1 |
| **m2-nm17**  **DQ519568** |  | 1 |  |  |  |  |  |  |  |  |  |  |  |  | 1 |
| **m2-nm18**  **DQ519567** |  |  |  | 1 |  |  |  |  |  |  |  |  |  |  | 1 |
| **Total** | 8 | 23 | 15 | 1 | 1 | 4 | 1 | 21 | 3 | 1 | 6 | 3 | 1 | 1 | 89 |

The abbreviation “nm” stands for “no matches”.

**Table S5: Distribution of the *16S rRNA* variants in the different animal species (sequences derived in own study and from GenBank)**

| **Host**  **Variant**  **exemplary Accession** | **Human** | **Dog** | **Horse** | **Cat** | **Hedgehog** | **Red fox** | **Cattle** | **small ruminants** | **Bison** | **Moose** | **Roe deer** | **Red deer** | **Sika deer** | **Fallow deer** | **Water deer** | **Mouflon** | **Chamois** | **Ibex** | **Wild boar** | **Rodent** | **Total** |
| --- | --- | --- | --- | --- | --- | --- | --- | --- | --- | --- | --- | --- | --- | --- | --- | --- | --- | --- | --- | --- | --- |
| **16S-1(A)**  **KU705194** | 1 | 45 | 1 | 3 | 43 | 5 |  |  |  |  |  |  |  |  |  |  |  |  |  |  | 98 |
| **16S-2(B)**  **KU705181** | 43 | 66 | 42 | 1 | 1 | 3 |  | 6 |  |  | 2 | 8 | 3 | 2 |  | 3 |  |  | 16 | 3 | 199 |
| **16S-3(D)**  **JF893931** |  |  | 1 |  |  |  |  |  |  |  | 9 |  |  |  |  |  |  |  |  |  | 10 |
| **16S-7(I)**  **KU705184** |  |  |  |  |  |  |  |  |  |  | 15 |  | 1 |  |  |  |  |  |  |  | 16 |
| **16S-8(J)**  **KU705187** |  |  |  |  |  |  |  |  |  |  |  | 1 | 1 |  |  |  |  |  |  |  | 2 |
| **16S-9(K)**  **FJ829792** |  | 1 |  |  |  |  |  |  |  |  |  |  |  |  |  |  |  |  |  |  | 1 |
| **16S-10(L)**  **JN656383** |  | 1 |  |  |  |  |  |  |  |  |  |  |  |  |  |  |  |  |  |  | 1 |
| **16S-12(N)**  **JX627361** |  |  |  |  |  |  |  |  |  |  | 21 |  |  |  |  |  |  |  |  |  | 21 |
| **16S-13(O)**  **KU705116** |  |  |  | 1 |  |  |  |  |  |  | 13 |  |  |  |  |  |  |  |  |  | 14 |
| **16S-16(S)**  **KU705117** |  |  | 1 |  | 1 |  |  | 7 |  |  | 1 | 21 | 4 |  |  | 5 | 2 | 2 | 1 | 3 | 48 |
| **16S-17(T)**  **FJ812398** |  |  |  |  |  |  |  |  |  |  |  | 1 |  |  |  |  |  |  |  |  | 1 |
| **16S-19(V)**  **KU705153** |  |  |  |  | 1 |  |  | 1 |  |  | 3 |  |  | 1 |  |  |  |  | 2 | 2 | 10 |
| **16S-20(W)**  **KU705119** | 5 | 1 | 2 |  | 1 |  | 42 | 14 (1x goat) | 14 |  | 5 | 8 | 4 | 1 | 1 | 8 | 9 |  | 1 | 32 | 148 |
| **16S-21(X)**  **KU705142** |  |  |  |  |  |  | 1 | 3 (2x goat) |  |  | 23 |  | 1 | 1 |  | 1 |  |  | 1 |  | 31 |
| **16S-22(Y)**  **KU705143** |  |  |  |  |  |  | 1 | 1 (goat) |  | 1 | 22 |  |  | 1 |  | 2 |  |  |  |  | 28 |
| **16S-23(Z)**  **OQ869753** |  |  |  |  |  |  |  | 1 (goat) |  |  |  |  |  |  |  | 1 | 1 |  |  |  | 3 |
| **16S-24**  **KU705147** |  | 1 |  |  |  |  |  |  |  |  | 1 |  |  |  |  |  |  |  |  |  | 2 |
| **16S-25**  **KU705130** |  |  |  |  |  |  |  |  |  |  |  | 1 |  |  |  |  |  |  |  |  | 1 |
| **16S-26**  **KU705139** |  |  |  |  |  |  |  |  |  |  |  | 1 |  |  |  |  |  |  |  |  | 1 |
| **16S-27**  **KU705195** |  |  |  |  |  | 1 |  |  |  |  |  |  |  |  |  |  |  |  |  |  | 1 |
| **16S-28**  **KU705123** |  |  |  |  |  |  |  |  |  |  |  | 1 |  |  |  |  |  |  |  |  | 1 |
| **16S-29**  **KU705183** |  |  |  |  |  |  |  |  |  |  |  |  | 1 |  |  |  |  |  |  |  | 1 |
| **16S-30**  **KU705126** |  |  |  |  |  |  |  |  |  |  |  | 1 |  |  |  |  |  |  |  | 3 | 4 |
| **16S-nm1 DQ458808** |  |  |  |  |  |  |  |  |  |  |  |  |  |  |  |  |  |  |  | 13 | 13 |
| **16S-nm2** **GU556622** |  |  |  |  |  |  |  |  |  |  |  |  |  |  | 2 |  |  |  |  |  | 2 |
| **16S-nm3**  **AY741099** |  | 2 |  |  |  |  |  |  |  |  |  |  |  |  |  |  |  |  |  |  | 2 |
| **16S-nm4**  **EU781707** |  | 2 |  |  |  |  |  |  |  |  |  |  |  |  |  |  |  |  |  |  | 2 |
| **16S-nm5**  **KC740440** |  |  |  |  |  |  |  |  |  |  |  |  |  |  |  |  |  |  |  | 5 | 5 |
| **16S-nm6**  **KC740435** |  |  |  |  |  |  |  |  |  |  |  |  |  |  |  |  |  |  |  | 2 | 2 |
| **16S-nm7** **GU236573** |  |  |  |  |  |  |  |  |  |  | 2 |  |  |  |  |  |  |  |  |  | 2 |
| **16S-nm8** **GU236568** |  |  |  |  |  |  |  |  |  |  | 2 |  |  |  |  |  |  |  |  |  | 2 |
| **16S-nm9**  **GU236577** |  |  |  |  |  |  |  |  |  |  |  | 1 |  |  |  |  |  |  |  |  | 1 |
| **16S-nm10**  **KC800985** |  |  |  |  |  |  |  |  |  | 1 |  |  |  |  |  |  |  |  |  |  | 1 |
| **16S-nm11**  **GU391318** |  |  |  |  |  |  |  |  |  |  |  |  |  |  |  |  |  |  | 1 |  | 1 |
| **16S-nm12**  **GU391317** |  |  |  |  |  |  |  |  |  |  |  |  |  |  |  |  |  |  | 1 |  | 1 |
| **16S-nm13**  **GU556625** |  |  |  |  |  |  |  |  |  |  |  |  |  |  | 1 |  |  |  |  |  | 1 |
| **16S-nm14**  **GU556623** |  |  |  |  |  |  |  |  |  |  |  |  |  |  | 1 |  |  |  |  |  | 1 |
| **16S-nm15**  **HQ872464** |  |  |  |  |  |  |  | 1 |  |  |  |  |  |  |  |  |  |  |  |  | 1 |
| **16S-nm16**  **DQ458806** |  |  |  |  |  |  |  |  |  |  |  |  |  |  |  |  |  |  |  | 1 | 1 |
| **16S-nm17**  **AY741095** |  | 1 |  |  |  |  |  |  |  |  |  |  |  |  |  |  |  |  |  |  | 1 |
| **16S-nm18**  **KF576219** |  | 1 |  |  |  |  |  |  |  |  |  |  |  |  |  |  |  |  |  |  | 1 |
| **16S-nm19**  **AF172164** |  |  | 1 |  |  |  |  |  |  |  |  |  |  |  |  |  |  |  |  |  | 1 |
| **16S-nm20**  **GU236706** |  | 1 |  |  |  |  |  |  |  |  |  |  |  |  |  |  |  |  |  |  | 1 |
| **16S-nm21 GU236698** |  | 1 |  |  |  |  |  |  |  |  |  |  |  |  |  |  |  |  |  |  | 1 |
| **16S-nm22**  **GU236645** |  |  |  |  |  |  |  | 1 |  |  |  |  |  |  |  |  |  |  |  |  | 1 |
| **16S-nm23**  **KC740432** |  |  |  |  |  |  |  |  |  |  |  |  |  |  |  |  |  |  |  | 1 | 1 |
| **16S-nm24**  **KC740441** |  |  |  |  |  |  |  |  |  |  |  |  |  |  |  |  |  |  |  | 1 | 1 |
| **16S-nm25 KC740431** |  |  |  |  |  |  |  |  |  |  |  |  |  |  |  |  |  |  |  | 1 | 1 |
| **16S-nm26 GU236574** |  |  |  |  |  |  |  |  |  |  | 1 |  |  |  |  |  |  |  |  |  | 1 |
| **16S-nm27**  **GU236566** |  |  |  |  |  |  |  |  |  |  | 1 |  |  |  |  |  |  |  |  |  | 1 |
| **16S-nm28 GU236541** |  |  |  |  |  |  |  |  |  |  | 1 |  |  |  |  |  |  |  |  |  | 1 |
| **Total** | 49 | 123 | 48 | 5 | 47 | 9 | 44 | 35 | 14 | 2 | 122 | 44 | 15 | 6 | 5 | 20 | 12 | 2 | 23 | 67 | 692 |

The abbreviation “nm” stands for “no matches”.

**Table S6: Distribution of the *msp4* variants in sequences obtained from own study and the GenBank**

| **Host**  **Variant**  **exemplary Accession** | **Human** | **Dog** | **Horse/**  **Donkey** | **Cat** | **Hedgehog** | **Red fox** | **Cattle** | **Small ruminants** | **Bison** | **Roe deer** | **Red deer** | **Sika deer** | **Fallow deer** | **Reindeer** | **Mouflon** | **Chamois** | **Ibex** | **Rodent** | **Total** |
| --- | --- | --- | --- | --- | --- | --- | --- | --- | --- | --- | --- | --- | --- | --- | --- | --- | --- | --- | --- |
| **m4-1(A)**  **HM028676** |  |  |  |  |  |  |  | 2 (goat) |  |  |  |  |  |  |  |  |  |  | 2 |
| **m4-2(B/C)**  **KU712134** |  | 19 | 12 | 1 |  |  |  | 2 (goat) |  | 2 |  |  |  |  |  |  |  |  | 36 |
| **m4-3(D)**  **OQ866966** |  | 1 | 1 |  |  |  |  |  |  |  |  |  |  |  |  |  |  |  | 2 |
| **m4-6(E)**  **GU265830** |  |  |  |  |  |  |  |  |  |  | 1 |  |  |  |  |  |  |  | 1 |
| **m4-7(F)**  **KU712155** |  |  |  |  |  |  |  |  |  |  | 2 |  |  |  |  |  |  |  | 2 |
| **m4-9(G)**  **GU265833** |  |  |  |  |  |  |  |  |  |  | 1 |  |  |  |  |  |  |  | 1 |
| **m4-8(H)**  **KU712149** |  |  |  |  |  |  |  | 1 |  |  | 1 |  |  |  | 1 |  |  |  | 3 |
| **m4-4(J)**  **GU265828** |  |  |  |  |  |  |  |  |  |  | 1 |  |  |  |  |  |  |  | 1 |
| **m4-5(I)**  **KU712150** |  |  |  |  |  |  |  | 5 |  |  |  |  |  |  | 4 | 2 |  |  | 11 |
| **m4-10(K)**  **GU265837** |  |  |  |  |  |  |  |  |  |  |  |  |  |  |  | 2 |  |  | 2 |
| **m4-12(M)**  **GU265835** |  |  |  |  |  |  |  |  |  | 2 |  |  |  |  |  |  |  |  | 2 |
| **m4-13(N)**  **KU712165** |  | 1 |  |  |  |  | 1 |  |  | 18 |  | 1 |  | 1 |  |  | 2 |  | 24 |
| **m4-14(O)**  **OQ866977** |  |  |  |  |  |  | 9 |  |  |  |  |  |  |  |  |  |  |  | 9 |
| **m4-15(P)**  **HM028677** |  |  |  |  |  |  | 2 |  |  |  |  |  |  |  |  |  |  |  | 2 |
| **m4-16(Q)**  **HM028678** |  |  |  |  |  |  | 3 |  |  |  |  |  |  |  |  |  |  |  | 3 |
| **m4-17(R)**  **HM028679** |  |  |  |  |  |  | 1 |  |  |  |  |  |  |  |  |  |  |  | 1 |
| **m4-18(S)**  **JF893893** |  |  | 1 |  |  |  |  | 1 |  |  |  |  |  |  |  |  |  |  | 2 |
| **m4-20**  **KU712135** |  | 9 |  |  | 18 | 3 |  | 3 |  |  |  |  |  |  |  |  |  |  | 33 |
| **m4-21**  **KU712148** |  |  |  |  |  |  |  |  |  |  |  | 2 |  |  | 1 |  |  |  | 3 |
| **m4-22**  **OQ866992** |  |  |  |  |  |  |  |  |  |  |  |  |  |  | 1 |  |  |  | 1 |
| **m4-23**  **KU712157** |  |  |  |  |  |  |  |  |  | 3 | 1 |  |  |  |  |  |  |  | 4 |
| **m4-24**  **KU712156** |  |  |  |  |  |  |  |  |  |  | 1 |  |  |  |  |  |  |  | 1 |
| **m4-25**  **KU712158** |  |  |  |  |  |  |  |  |  |  | 1 |  |  |  |  |  |  |  | 1 |
| **m4-26**  **KU712159** |  |  |  |  |  |  |  |  |  |  | 1 |  |  |  |  |  |  |  | 1 |
| **m4-27**  **KU712160** |  |  |  |  |  |  |  |  |  |  | 1 |  |  |  |  |  |  |  | 1 |
| **m4-28**  **KU712161** |  |  |  |  |  |  |  |  |  |  | 1 |  |  |  |  |  |  |  | 1 |
| **m4-29**  **KU712162** |  |  |  |  |  |  |  |  |  |  | 2 |  |  |  |  |  |  |  | 2 |
| **m4-31**  **KU712178** |  |  |  |  |  |  |  |  |  |  |  | 1 |  |  |  |  |  |  | 1 |
| **m4-32**  **KU712183** |  |  |  |  |  |  |  |  |  |  |  | 1 |  |  |  |  |  |  | 1 |
| **m4-33**  **KU712185** |  |  |  |  |  |  |  |  |  |  |  | 1 |  |  |  |  |  |  | 1 |
| **m4-34**  **KU712187** |  |  |  |  |  |  |  |  |  |  |  | 1 |  |  |  |  |  |  | 1 |
| **m4-35**  **KU712174** |  |  |  |  |  |  |  |  |  | 2 |  |  |  |  |  |  |  |  | 2 |
| **m4-36**  **KU712144** |  |  |  |  |  |  |  |  |  |  |  |  | 3 |  |  |  |  |  | 3 |
| **m4-37**  **KU712152** |  |  |  |  |  |  |  | 1 |  |  | 1 |  |  |  |  |  |  |  | 2 |
| **m4-38**  **KU712153** |  |  |  |  |  |  |  |  |  |  | 1 |  |  |  |  |  |  |  | 1 |
| **m4-39**  **KU712171** |  |  |  |  |  |  |  |  |  | 1 |  |  |  |  |  |  |  |  | 1 |
| **m4-40**  **KU712147** |  |  |  |  |  |  |  |  |  |  |  |  | 1 |  |  |  |  |  | 1 |
| **m4-41**  **KU712175** |  |  |  |  |  |  |  |  |  | 1 |  |  |  |  |  |  |  |  | 1 |
| **m4-42**  **KU712166** |  |  |  |  |  |  |  |  |  | 1 |  |  |  |  |  |  |  |  | 1 |
| **m4-43**  **KU712154** |  |  |  |  |  |  |  |  |  |  | 1 |  |  |  |  |  |  |  | 1 |
| **m4-44**  **KU712180** |  |  |  |  |  |  |  |  |  |  | 1 | 1 |  |  |  |  |  |  | 2 |
| **m4-45**  **KU712182** |  |  |  |  |  |  |  |  |  | 1 |  | 1 |  |  |  |  |  |  | 2 |
| **m4-46**  **KU712184** |  |  |  |  |  |  |  |  |  |  |  | 1 |  |  |  |  |  |  | 1 |
| **m4-47**  **KU712186** |  |  |  |  |  |  |  |  |  |  |  | 1 |  |  |  |  |  |  | 1 |
| **m4-48**  **KU712177** |  |  |  |  |  |  |  |  |  | 1 |  |  |  |  |  |  |  |  | 1 |
| **m4-49**  **KU587073** |  |  |  |  |  |  | 12 |  |  |  |  |  |  |  |  |  |  |  | 12 |
| **m4-50**  **KU587076** |  |  |  |  |  |  | 3 |  |  |  |  |  |  |  |  |  |  |  | 3 |
| **m4-51**  **EU857665** |  |  |  |  |  |  | 6 |  | 2 |  |  |  |  |  |  |  |  |  | 8 |
| **m4-nm1**  **KF420102** |  |  |  |  |  |  |  |  |  |  |  |  |  |  |  |  |  | 11 | 11 |
| **m4-nm2**  **EU857674** | 2 |  |  |  |  |  |  |  |  |  |  |  |  |  |  |  |  |  | 2 |
| **m4-nm3**  **GQ412348** |  |  |  |  |  |  |  |  |  |  |  |  |  |  |  |  |  | 3 | 3 |
| **m4-nm4**  **EU857670** |  |  |  |  |  |  | 1 |  |  |  |  |  |  |  |  |  |  |  | 1 |
| **m4-nm5**  **EU857669** |  |  |  |  |  |  | 1 |  |  |  |  |  |  |  |  |  |  |  | 1 |
| **m4-nm6**  **EU857666** |  |  |  |  |  |  | 1 |  |  |  |  |  |  |  |  |  |  |  | 1 |
| **m4-nm7**  **JN005728** |  |  |  |  |  |  |  |  |  | 1 |  |  |  |  |  |  |  |  | 1 |
| **m4-nm8**  **JN005726** |  |  |  |  |  |  |  |  |  | 1 |  |  |  |  |  |  |  |  | 1 |
| **m4-nm9 EU008082** |  |  |  |  |  |  |  |  |  |  |  |  |  |  |  |  |  | 1 | 1 |
| **m4-nm10**  **AY706391** |  |  |  |  |  |  |  | 1 |  |  |  |  |  |  |  |  |  |  | 1 |
| **m4-nm11**  **AY706388** |  |  |  |  |  |  |  |  | 1 |  |  |  |  |  |  |  |  |  | 1 |
| **m4-nm12**  **AY702925** |  |  | 1 |  |  |  |  |  |  |  |  |  |  |  |  |  |  |  | 1 |
| **m4-nm13**  **EU436164** |  |  |  |  |  |  |  | 1 |  |  |  |  |  |  |  |  |  |  | 1 |
| **m4-nm14**  **EU180065** |  |  |  |  |  |  |  |  |  |  | 1 |  |  |  |  |  |  |  | 1 |
| **m4-nm15**  **EU180064** |  |  |  |  |  |  |  |  |  |  | 1 |  |  |  |  |  |  |  | 1 |
| **m4-nm16**  **EU180063** |  |  |  |  |  |  |  |  |  |  | 1 |  |  |  |  |  |  |  | 1 |
| **m4-nm17**  **EU180062** |  |  |  |  |  |  |  |  |  | 1 |  |  |  |  |  |  |  |  | 1 |
| **m4-nm18**  **EU180059** |  |  |  |  |  |  |  |  |  |  | 1 |  |  |  |  |  |  |  | 1 |
| **m4-nm19**  **EU180058** |  |  |  |  |  |  |  |  |  |  | 1 |  |  |  |  |  |  |  | 1 |
| **m4-nm20**  **AY829457** |  |  |  |  |  |  |  |  |  | 1 |  |  |  |  |  |  |  |  | 1 |
| **m4-nm21**  **AY829455** |  |  |  |  |  |  | 1 |  |  |  |  |  |  |  |  |  |  |  | 1 |
| **m4-nm22**  **JQ522935** |  |  |  |  |  |  |  | 1 |  |  |  |  |  |  |  |  |  |  | 1 |
| **Total** | 2 | 30 | 15 | 1 | 18 | 3 | 41 | 18 | 3 | 36 | 23 | 11 | 4 | 1 | 7 | 4 | 2 | 15 | 234 |

The abbreviation “nm” stands for “no matches”. **Table S7: Distribution of the *groEL* variants in the different animal species (sequences derived in own study and from GenBank)**

| **Host**  **Variant**  **exemplary Accession** | **Human** | **Dog** | **Horse** | **Cat** | **Hedgehog** | **Red fox** | **Cattle** | **Small ruminants** | **Moose** | **Cervid** | **Roe deer** | **Red deer** | **Sika deer** | **Fallow deer** | **Water deer** | **Mouflon** | **Chamois** | **Ibex** | **Wild boar** | **Rodent** | **Total** |
| --- | --- | --- | --- | --- | --- | --- | --- | --- | --- | --- | --- | --- | --- | --- | --- | --- | --- | --- | --- | --- | --- |
| **g-1(A)**  **JN656294** |  | 15 | 1 |  | 27 | 2 |  |  |  |  | 1 |  |  |  |  |  |  |  |  |  | 46 |
| **g-2(B)**  **JN656295** | 1 | 21 | 12 | 1 |  | 1 |  | 2 |  |  |  |  |  |  |  |  |  |  | 1 |  | 39 |
| **g-3(C) OQ867037** |  |  |  |  |  |  | 9 |  |  |  | 1 |  |  |  |  |  |  |  |  |  | 10 |
| **g-4(D) GQ452226** |  |  |  |  |  |  |  | 2 (goat) |  |  | 2 |  |  |  |  |  |  |  |  |  | 4 |
| **g-5(E) GQ452225** |  |  |  |  |  |  |  | 1 (goat) |  | 1 | 2 |  |  |  |  |  |  |  |  |  | 4 |
| **g-6(F) GQ988763** |  |  |  |  |  |  |  |  |  |  | 6 | 2 |  |  |  |  |  | 2 |  |  | 10 |
| **g-7(G) GQ988761** |  |  |  |  |  |  |  |  |  | 1 | 15 | 2 |  |  |  |  |  |  |  |  | 18 |
| **g-8(H) GQ988765** |  |  |  |  |  |  |  | 1 |  |  | 1 | 1 |  | 2 |  | 3 | 1 |  |  |  | 9 |
| **g-9(I) GQ988768** |  |  |  |  |  |  |  |  |  |  |  |  |  |  |  |  | 1 |  |  |  | 1 |
| **g-10(J) GQ988769** |  |  |  |  |  |  |  |  |  |  |  |  |  |  |  |  | 1 |  |  |  | 1 |
| **g-12(K) GQ988754** |  |  | 2 |  |  |  |  |  |  |  | 2 |  |  |  |  |  |  |  |  |  | 4 |
| **g-13(L) JF893918** |  |  |  |  |  |  |  | 1 |  |  |  | 1 | 1 |  |  | 2 |  |  |  |  | 5 |
| **g-14(M)** **GQ988758** |  |  |  |  |  |  |  |  |  |  |  | 1 |  |  |  |  |  |  |  |  | 1 |
| **g-15(N)** **GQ988766** |  |  |  |  |  |  | 3 |  |  |  |  |  |  |  |  |  |  |  |  |  | 3 |
| **g-16(O) GQ988756** |  |  |  |  |  |  | 1 |  |  |  |  |  |  |  |  |  | 1 |  |  |  | 2 |
| **g-18(X) GQ452232** |  |  |  |  |  |  | 11 | 2 |  |  |  |  |  |  |  |  |  |  |  |  | 13 |
| **g-19(Y)** **GQ452231** |  |  |  |  |  |  | 1 |  |  |  |  |  |  |  |  |  |  |  |  |  | 1 |
| **g-20 KU712112** |  |  |  |  |  |  |  |  | 2 |  | 3 |  |  |  |  |  |  |  |  |  | 5 |
| **g-21** **KU712100** |  |  |  |  |  |  |  |  |  |  |  |  |  |  |  | 2 |  |  |  |  | 2 |
| **g-22**  **KU712098** |  |  |  |  |  |  |  |  |  |  |  |  |  |  |  | 1 |  |  |  |  | 1 |
| **g-23** **KU712105** |  |  |  |  |  |  |  |  |  |  |  |  |  |  |  | 1 |  |  |  |  | 1 |
| **g-24** **KU712093** |  |  |  |  |  |  |  | 2 |  |  | 1 |  | 1 | 1 |  |  |  |  |  |  | 5 |
| **g-25** **OQ867048** |  |  |  |  |  |  |  |  |  |  | 1 |  |  |  |  |  |  |  |  |  | 1 |
| **g-26 KU712123** |  |  |  |  |  |  |  |  |  |  | 1 |  |  |  |  |  |  |  |  |  | 1 |
| **g-27** **OQ867047** |  |  |  |  |  |  |  |  |  |  |  |  |  |  |  |  | 1 |  |  |  | 1 |
| **g-28 KU712127** |  |  |  |  |  |  |  |  |  |  |  |  | 1 |  |  |  |  |  |  |  | 1 |
| **g-29**  **KU712097** |  |  |  |  |  |  |  |  |  |  |  |  |  | 1 |  |  |  |  |  |  | 1 |
| **g-30** **KU712114** |  |  |  |  |  |  |  |  |  |  | 1 |  |  |  |  |  |  |  |  |  | 1 |
| **g-31** **KU712117** |  |  |  |  |  |  |  |  |  |  | 1 |  |  |  |  |  |  |  |  |  | 1 |
| **g-32 KU712107** |  |  |  |  |  |  |  |  |  |  |  | 1 |  |  |  |  |  |  |  |  | 1 |
| **g-33** **KU712108** |  |  |  |  |  |  |  |  |  |  |  | 1 |  |  |  |  |  |  |  |  | 1 |
| **g-34** **KU712125** |  |  |  |  |  |  |  |  |  |  |  |  | 1 |  |  | 1 |  |  |  |  | 2 |
| **g-35 KU712126** |  |  |  |  |  |  |  | 4 |  |  | 1 |  | 1 |  |  |  |  |  |  |  | 6 |
| **g-nm1** **KF383231** |  |  |  |  |  |  |  |  |  |  |  |  |  |  |  |  |  |  |  | 6 | 6 |
| **g-nm2** **KC583432** |  |  |  |  |  |  |  |  |  |  |  |  |  |  |  |  |  |  |  | 3 | 3 |
| **g-nm3** **EU860090** | 4 | 1 | 5 | 1 |  |  |  |  |  |  |  |  |  |  |  |  |  |  |  | 5 | 16 |
| **g-nm4** **JF494836** |  |  |  |  |  |  |  |  |  |  |  |  |  |  |  |  |  |  |  | 2 | 2 |
| **g-nm5** **JN005748** |  |  |  |  |  |  |  |  |  | 1 | 1 |  |  |  |  |  |  |  |  |  | 2 |
| **g-nm6** **JN005747** |  |  |  |  |  |  |  |  |  |  | 3 |  |  |  |  |  |  |  |  |  | 3 |
| **g-nm7** **HQ630619** |  |  |  |  |  |  |  |  |  |  |  |  |  |  |  |  |  |  |  | 2 | 2 |
| **g-nm8** **HQ630615** |  |  |  |  |  |  |  |  |  |  |  |  |  |  |  |  |  |  |  | 2 | 2 |
| **g-nm9** **AF478561** |  |  |  |  |  |  |  |  |  |  | 2 |  |  |  |  |  |  |  |  |  | 2 |
| **g-nm10** **KC800986** |  |  |  |  |  |  |  |  | 1 |  |  |  |  |  |  |  |  |  |  |  | 1 |
| **g-nm11** **KC583433** |  |  |  |  |  |  |  |  |  |  |  |  |  |  |  |  |  |  |  | 1 | 1 |
| **g-nm12** **KC583431** |  |  |  |  |  |  |  |  |  |  |  |  |  |  |  |  |  |  |  | 1 | 1 |
| **g-nm13** **KC753762** |  |  |  |  |  |  |  |  |  |  |  |  |  |  |  |  |  |  |  | 1 | 1 |
| **g-nm14** **EU860088** |  |  |  |  |  |  |  | 1 |  |  |  |  |  |  |  |  |  |  |  |  | 1 |
| **g-nm15** **JF494838** |  |  |  |  |  |  |  |  |  |  |  |  |  |  |  |  |  |  |  | 1 | 1 |
| **g-nm16** **JF494833** |  | 1 |  |  |  |  |  |  |  |  |  |  |  |  |  |  |  |  |  |  | 1 |
| **g-nm17** **HM752098** |  |  |  |  |  |  |  |  |  |  |  |  |  |  | 1 |  |  |  |  |  | 1 |
| **g-nm18** **AY848752** |  | 1 |  |  |  |  |  |  |  |  |  |  |  |  |  |  |  |  |  |  | 1 |
| **g-nm19** **AY848751** |  | 1 |  |  |  |  |  |  |  |  |  |  |  |  |  |  |  |  |  |  | 1 |
| **g-nm20** **AY848750** |  | 1 |  |  |  |  |  |  |  |  |  |  |  |  |  |  |  |  |  |  | 1 |
| **g-nm21** **AY848749** |  |  | 1 |  |  |  |  |  |  |  |  |  |  |  |  |  |  |  |  |  | 1 |
| **g-nm22** **AY848748** |  |  | 1 |  |  |  |  |  |  |  |  |  |  |  |  |  |  |  |  |  | 1 |
| **g-nm23** **AY848747** |  |  | 1 |  |  |  |  |  |  |  |  |  |  |  |  |  |  |  |  |  | 1 |
| **g-nm24** **EU157920** |  |  |  |  |  |  |  |  |  | 1 |  |  |  |  |  |  |  |  |  |  | 1 |
| **g-nm25** **AF548386** |  |  |  |  |  |  |  | 1 |  |  |  |  |  |  |  |  |  |  |  |  | 1 |
| **g-nm26** **EF647585** |  |  | 1 |  |  |  |  |  |  |  |  |  |  |  |  |  |  |  |  |  | 1 |
| **g-nm27** **AY220469** |  |  |  |  |  |  |  |  |  |  | 1 |  |  |  |  |  |  |  |  |  | 1 |
| **g-nm28** **AY220468** |  |  |  |  |  |  |  |  |  |  | 1 |  |  |  |  |  |  |  |  |  | 1 |
| **g-nm29** **AF478563** |  |  |  |  |  |  |  |  |  |  | 1 |  |  |  |  |  |  |  |  |  | 1 |
| **g-nm30** **AF478556** |  |  |  |  |  |  |  |  |  |  | 1 |  |  |  |  |  |  |  |  |  | 1 |
| **g-nm31** **AF478554** |  |  |  |  |  |  |  |  |  |  | 1 |  |  |  |  |  |  |  |  |  | 1 |
| **g-nm32** **JN055360** |  |  |  |  |  |  |  |  |  |  |  |  | 1 |  |  |  |  |  |  |  | 1 |
| **g-nm33** **JN055359** |  |  |  |  |  |  |  |  |  |  |  |  | 1 |  |  |  |  |  |  |  | 1 |
| **g-nm34** **AF478553** |  |  |  |  |  |  |  |  |  |  | 1 |  |  |  |  |  |  |  |  |  | 1 |
| **Total** | 5 | 41 | 24 | 2 | 27 | 3 | 25 | 17 | 3 | 4 | 51 | 9 | 7 | 4 | 1 | 10 | 5 | 2 | 1 | 24 | 265 |

The abbreviation “nm” stands for “no matches”.

1. Massung RF, Slater K, Owens JH, Nicholson WL, Mather TN, Solberg VB, et al. Nested PCR assay for detection of granulocytic ehrlichiae. J Clin Microbiol. 1998;36 4:1090-5.

2. Courtney JW, Kostelnik LM, Zeidner NS, Massung RF. Multiplex real-time PCR for detection of Anaplasma phagocytophilum and Borrelia burgdorferi. J Clin Microbiol. 2004;42 7:3164-8; doi: 10.1128/Jcm.42.7.3164-3168.2004.

3. Lin Q, Rikihisa Y, Felek S, Wang X, Massung RF, Woldehiwet Z. Anaplasma phagocytophilum has a functional msp2 gene that is distinct from p44. Infect Immun. 2004;72 7:3883-9; doi: 10.1128/IAI.72.7.3883-3889.2004.

4. de la Fuente J, Massung RF, Wong SJ, Chu FK, Lutz H, Meli M, et al. Sequence analysis of the msp4 gene of Anaplasma phagocytophilum strains. J Clin Microbiol. 2005;43 3:1309-17; doi: 10.1128/JCM.43.3.1309-1317.2005.

5. Bown KJ, Lambin X, Ogden NH, Petrovec M, Shaw SE, Woldehiwet Z, et al. High-resolution genetic fingerprinting of European strains of Anaplasma phagocytophilum by use of multilocus variable-number tandem-repeat analysis. J Clin Microbiol. 2007;45 6:1771-6; doi: 10.1128/JCM.00365-07.

6. Alberti A, Addis MF, Sparagano O, Zobba R, Chessa B, Cubeddu T, et al. Anaplasma phagocytophilum, Sardinia, Italy. Emerg Infect Dis. 2005;11 8:1322-4; doi: 10.3201/eid1108.050085.

7. Alberti A, Zobba R, Chessa B, Addis MF, Sparagano O, Pinna Parpaglia ML, et al. Equine and canine Anaplasma phagocytophilum strains isolated on the island of Sardinia (Italy) are phylogenetically related to pathogenic strains from the United States. Appl Environ Microbiol. 2005;71 10:6418-22; doi: 10.1128/AEM.71.10.6418-6422.2005.
